# Multi-stakeholder perspectives on urban regeneration: a spatial gradient analysis of heat and pollution effects

**DOI:** 10.1007/s00484-025-02894-8

**Published:** 2025-03-24

**Authors:** Jingqi Zhang, Xinyue Gu

**Affiliations:** 1 Faculty of Science, Hong Kong Baptist University, Hong Kong, China; 2 Department of Land Surveying and Geo-Informatics, The Hong Kong Polytechnic University, 11 Yuk Choi Rd, Hung Hom, Hong Kong, China

**Keywords:** Urban heat, Urban pollution, Urban regeneration, Spatial gradient, Stakeholders

## Abstract

The acceleration of urbanization has caused severe environmental problems for cities, making the implementation of sustainable urban regeneration projects an important part of urban construction. Previous studies have shown that different actions taken by various stakeholder groups at different stages of urban regeneration can affect the outcomes of urban regeneration. However, few studies have investigated the environmental impacts on the urban regeneration plots and their surroundings during the urban regeneration implementation stage under the leadership of different stakeholders. Therefore, this study selected 12 plots under three urban regeneration models—government-led, government-enterprise cooperation-led, and multi-stakeholder cooperation-led—and examined the median or mean values of land surface temperature, air temperature, carbon monoxide concentration, nitrogen dioxide concentration, and sulfur dioxide concentration for each season over four years, from pre-regeneration to the implementation stage. It analyzed the urban heat and pollution effects within the plots and in multiple ring buffer zones of 50 m, 150 m, and 350 m outside the plots. The results indicated that: (1) The land surface temperature within the plots was significantly influenced by urban regeneration, while the impact on air quality was not as noticeable; (2) During winter, the urban heat and pollution within the plots were least affected by the implementation of urban regeneration; (3) Urban regeneration models with government involvement as the leading party were more likely to mitigate the impact of urban regeneration project implementation on urban heat and pollution. These findings are of significant importance for furthering the sustainability of urban regeneration.

**Supplementary Information:**

The online version contains supplementary material available at 10.1007/s00484-025-02894-8.

## Introduction

As the global urbanization process advances, many cities are confronted with a series of urban issues, including scarce land resources, environmental pollution, economic decline, and social conflicts. Urban regeneration is a crucial approach to addressing these urban problems and achieving sustainable urban development (Huang et al. 2020; Yıldız et al. 2020). When assessing the sustainability of urban regeneration projects, the focus is primarily on three dimensions: economic, social, and environmental. Among these three dimensions, the environmental dimension carries a high weight and can influence the economic dimension; therefore, a comprehensive emphasis on environmental sustainability is of great significance for the implementation of urban regeneration projects (Lin et al. 2021). Among the environmental dimensions, the issues of urban heat and air pollution are particularly prominent (Ulpiani 2021). Urban regeneration is often defined as the process of slum clearance and physical reconstruction in urban areas. However, the vision and actions of urban regeneration are more comprehensive and integrated, aimed at addressing multifaceted issues in urban areas and improving the economic, physical, social, and environmental conditions of impoverished regions (Lin et al. 2021). However, urban regeneration is a complex endeavour that involves many different stakeholders throughout the process. Different stakeholder groups, with varying preferences and interests, may choose different urban regeneration models and make distinct decisions that ultimately affect the outcome of urban regeneration (Zhou, Lan, and Zhou 2021). According to the main stakeholders including the government, developers, and residents, urban regeneration models can be categorized into three types: government-led, government-enterprise cooperation-led, and multi-stakeholder cooperation-led (Chu et al. 2020). The three urban regeneration models, driven by different interest preferences, exhibit different operational modes, as shown in Table 1. Therefore, urban regeneration is influenced by various complex conditions, and it is crucial to investigate and study the extent of the impact on urban heat and pollution issues during the urban regeneration process led by different stakeholders.

**Table 1:** The difference between three urban regeneration models

Urban regeneration models	Operational modes	Objective (interest preferences)
government-led	In an urban regeneration model where the government plays a dominant role in decision-making, characterized by strengthening governmental power and minimizing public participation, the government controls the progress of urban regeneration as well as land and taxation. It integrates resources, provides public services, and promotes cooperation among stakeholders to maximize economic benefits (Wang et al. 2022).	Local governments hope to: promote economic development, maintain social stability, improve the living environment, achieve “administrative and political” interests (Bai et al. 2023; Zhuang et al. 2019).
government-enterprise cooperation-led	Developers typically assume the role of project execution, applying for project approval from the government after obtaining the agreement of the majority of original property owners. Throughout this process, the government only plays a role in macro guidance and regulation. Urban regeneration projects involving developers often lead to higher expectations among property owners regarding compensation for their properties (Xie et al. 2024a, b; Xiao et al. 2022).	Developers hope to: achieve maximum benefit at the lowest cost, obtain direct profits, obtain long-term benefits, build a good brand image, pursue “marketing performance” benefits (Bai et al. 2023; Zhuang et al. 2019).
multi-stakeholder cooperation-led	It increases the proportion of public participation and involves the local community in the regeneration process, then emphasises community empowerment. Community planners act as a bridge between decision-makers and the public to convey information among different stakeholders, aiming to maximize benefits for all parties involved (Wang et al. 2021a, b).	Residents hope to: obtain compensation from land development, obtain social security benefits, improve their living environment and quality of life, achieve equity and justice, achieve “community” interests (Bai et al. 2023; Zhuang et al. 2019).

Existing research has shown that urban heat and air pollution are influenced by both natural and anthropogenic factors in their temporal and spatial distribution (Wang et al. 2021). Moreover, various variables of the built environment have a significant impact on the urban heat environment (Gu et al. 2024). Through specific urban regeneration strategies, air quality can be effectively improved and the urban heat island effect can be mitigated, for example, by redeveloping large vacant lots into long-term green infrastructure (Kim et al. 2020). However, it has been found that due to the complexity of urban regeneration, different types and stages of urban regeneration projects have varying effects on mitigating urban heat. The urban regeneration process can be divided into three stages: pre-regeneration, regeneration implementation, and post-regeneration. The pre-regeneration stage refers to the status of the area before the urban regeneration project begins. The regeneration implementation stage includes the demolition of existing buildings and the construction of new ones until the completion of the new structures. The post-regeneration stage is the period after the urban regeneration project is completed. During the regeneration implementation stage, a significant impact on urban heat was detected when dense mid-rise buildings, bare soil, and heavy industrial areas were transformed into open high-rise developments (S. Zheng, Chen, and Liu 2023; Liu et al. 2025). In addition, the urban heat effect exhibits a strong scale dependency, with existing research often focusing on large metropolitan areas and the city scale (Li et al. 2020). In cities with different levels of development or different geographical locations, the impact of urban form indicators on air quality varies, including area metrics, aggregation metrics, and shape metrics (Liang and Gong 2020).

The above research indicates that current studies on urban regeneration and issues of urban pollution and heat mainly focus on how urban regeneration can mitigate urban heat effects and air quality problems (Viecco et al. 2021; W. Wang and Shu 2020). Additionally, research on stakeholders in urban regeneration often focuses on the roles played by different stakeholders during the urban regeneration process and how to balance their relationships to achieve a win-win outcome (Zhu, Mu, and Liang 2022). However, the impact of urban regeneration on the environment may not be entirely positive, and there is still limited research on the climate change and urban air quality issues that can result from the activities of various city sectors during the urban regeneration process (Mi et al. 2019). There is also a lack of research on whether the involvement of different stakeholders during the implementation phase of urban regeneration affects the achievement of sustainability in urban regeneration projects (Yang 2014). To fill the research gap concerning the thermal and pollution effects during the urban regeneration implementation phase from the perspective of different stakeholders, this paper selects two indicators that are significant for the urban heat environment—land surface temperature (LST) and air temperature (Ta)—based on three urban regeneration models: government-led, government-enterprise cooperation-led, and multi-stakeholder cooperation-led (Cao et al. 2021), and the traditional air pollutants carbon monoxide (CO), nitrogen dioxide (NO_2_), and sulfur dioxide (SO_2_) (Ngarambe et al. 2021), an analysis of the thermal and pollution effects during the four-year urban regeneration process from winter 2018 to autumn 2022 was conducted for each of the 12 plots.

The main research question addressed in this study is the impact of urban regeneration, led by different stakeholders, on urban heat and pollution during the implementation phase. First, 12 plots in Changsha City, under the leadership of various stakeholders during the urban regeneration implementation phase, were selected, and temperature and air quality data were collected using remote sensing imagery combined with meteorological stations and ground observation stations. Second, GIS was used to process the raster data of all acquired plots, obtaining the median or average values of LST, Ta, CO, NO_2_, and SO_2_ for each quarter from winter 2018 to autumn 2022, and multi-ring buffer analyses were conducted around the periphery of each plot. Then, compare the changes in temperature and pollutant concentrations inside and outside the plots during the implementation of urban regeneration under different stakeholder-led urban regeneration models. Finally, based on the conclusions, policy recommendations were proposed to promote sustainable development during the urban regeneration implementation phase, aiming to provide a reference for selecting stakeholder participation and formulating policies to avoid urban heat and pollution during the implementation phase of urban regeneration projects.

## Materials and methods

### Research area

This study selected Changsha City, China (27°51’–28°41’N and 111°53’–114°15’E) as the research subject. It is located in the southern part of the Yangtze River region in central China, in the northeastern part of Hunan Province. The area has a subtropical monsoon climate. Additionally, Changsha has exhibited significant impacts from environmental issues such as high temperatures over many years (Xie et al. 2024a, b).

**Fig. 1:**
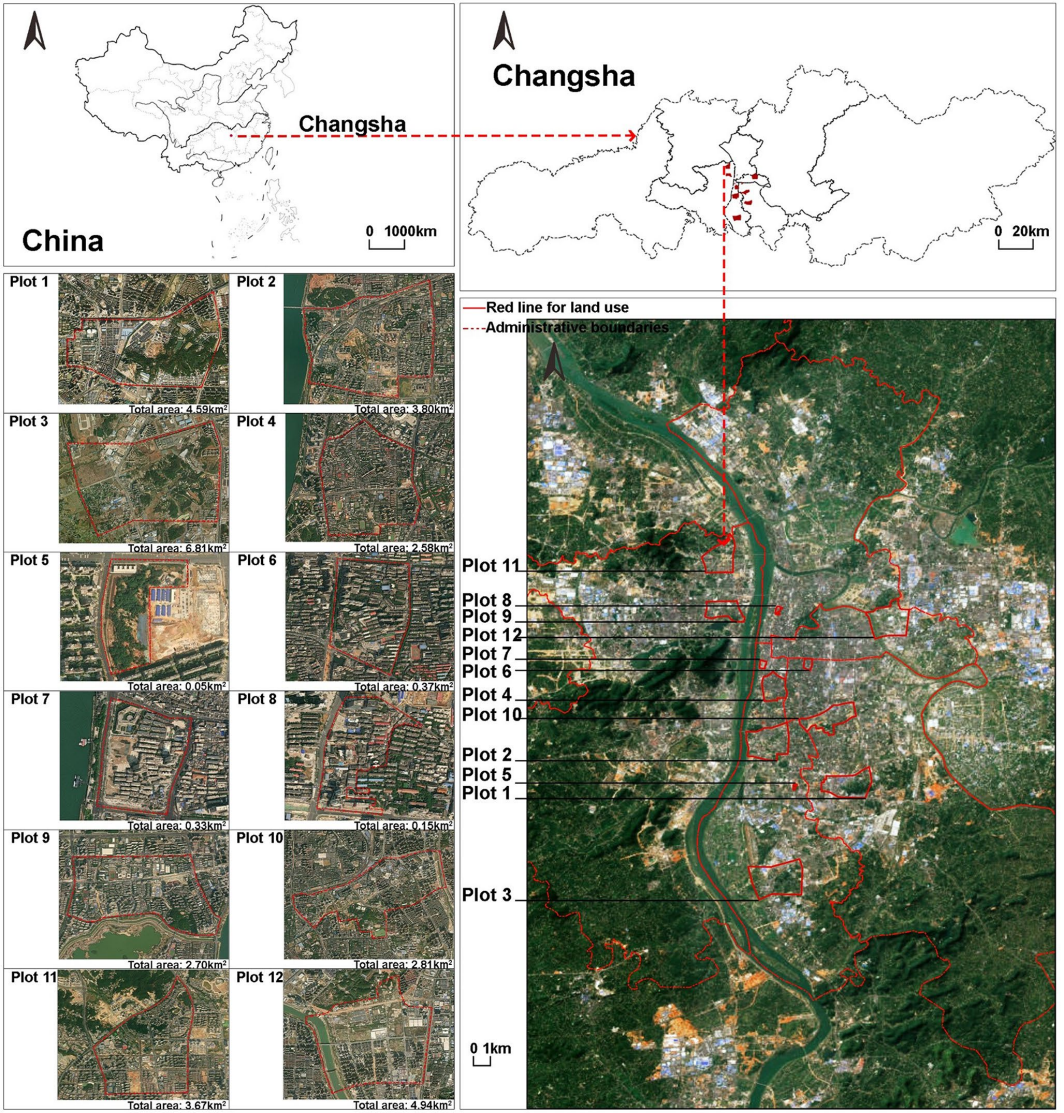
Location of the study area

For the three different urban regeneration models led by various stakeholders, four plots were selected as study areas for each model. According to official documents such as the Changsha City Urban Regeneration Special Plan published by the Changsha Municipal Government, a total of 12 plots that met the requirements were selected, as shown in Fig. 1. The urban regeneration projects in these study areas all started in 2020 or 2021. To ensure a better examination of the environmental impacts before and after the initiation of urban regeneration, the analysis covered four years from winter 2018 to autumn 2022, following the seasonal order of winter, spring, summer, and autumn. Special emphasis was placed on comparing the four seasons from winter 2018 to autumn 2019 with those from winter 2021 to autumn 2022, to assess the changes before and during the implementation phase of urban regeneration. The stages of urban regeneration corresponding to different plots over four years are shown in Supplementary Table 1. And through the resource and environmental science data registration and publication system, the annual NDVI and EVI 1KM datasets of China were obtained, providing the surface vegetation coverage conditions of the plots in 2018 and 2022, as shown in Supplementary Fig. 1 (Xu 2018). The continuous variable range of normalized difference vegetation index (NDVI) is from − 1 to 1, with a score of -1 indicating no vegetation and 1 indicating abundant healthy vegetation (Martinez and Labib 2023).

First, for the urban regeneration model led by government-enterprise cooperation, the plots are numbered 1–4. Secondly, for the urban regeneration model led by multi-stakeholder cooperation, the plots are numbered 5–8. Finally, for the urban regeneration model led by the government, the plots are numbered 9–12. The significant area differences among the twelve plots mainly exist between different urban regeneration models, while the plot areas within the same urban regeneration model are relatively similar. Given that large-scale demolition can have a greater environmental impact, these area differences might affect the comparison of environmental issues across plots under different urban regeneration models. However, for this study, which focuses on the environmental issues arising over time for each plot through urban regeneration and then compares these issues across different models, the influence of area differences is negligible.

### Data collection and processing

In this paper, the median or average values of the data for each season from winter 2018 to autumn 2022 were calculated for each of the 12 plots. Spring includes March, April, and May; summer includes June, July, and August; autumn includes September, October, and November; and winter includes December, the following year January and February. The specific processing procedures for different indicators are as follows:

#### Land surface temperature (LST) data

Land surface temperature describes the response of urban structures and surface materials to heat (Bai et al. 2024; Meng et al. 2022). The LST data used in this study were obtained by processing the LANDSAT/LC08/C02/T1_L2 dataset with Google Earth Engine, resulting in LST raster data with a spatial resolution of 30 m. The thermal infrared band ST_B10 directly corresponds to the LST and Celsius values of LST were acquired through code and formula conversion, as described by Eq. 1. Then, 273.15 was subtracted to convert from Kelvin to Celsius, as per Eq. 2, ultimately yielding the LST in degrees Celsius. During the process, Google Earth Engine was selected for data processing, and unwanted pixels were masked using the QA_PIXEL band. The median values for each season from winter 2018 to autumn 2022 were synthesized. The LST raster data for Changsha City over the specified period were then imported into ArcGIS 10.8 for further processing. Based on meteorological data provided by the National Centers for Environmental Information under the U.S. National Oceanic and Atmospheric Administration, outliers were removed by screening the average maximum and minimum temperatures for each season in Changsha, adding and subtracting 15 °C (Mutiibwa, Strachan, and Albright 2015), the incomplete data affected by cloud cover were completed using the Kriging interpolation method. Then, mask extraction was performed for each of the 12 plots. Finally, multi-ring buffers of 50 m, 150 m, and 350 m were established around the areas, and the average land surface temperature for all regions in each season was calculated.

1 $$\text{LST}\left(\text{K}\right)= \text{DN}\:0.00341802-149$$

2 $$\text{LST}\left(\circ\text{C}\right)=\text{LST}\left(\text{K}\right)-273.15$$

Where LST(K) is the generated land surface temperature in Kelvin, DN is the digital number from the thermal infrared band ST_B10 of the LANDSAT/LC08/C02/T1_L2 satellite image, and LST(℃) is the generated land surface temperature in degrees Celsius.

#### Air temperature (Ta) data

Air temperature data primarily describes the heat conditions within the atmosphere (Bai et al. 2024). The Ta data are sourced from the datasets shared by scholar Peng Shouzhang on the National Tibetan Plateau Data Center platform (Peng 2019), The spatial resolution is 1 km. In this study, the original data were processed, with units converted to degrees Celsius (℃), and the format was changed to raster (.tif) while extracting the data for Hunan Province. The resulting raster data were imported into ArcGIS 10.8 for processing. Using a three-month interval, the average Ta data for each quarter from December 2018 to November 2022 (covering winter, spring, summer, and autumn) were calculated using the raster calculator. Then, the data were clipped for each of the 12 plots, and multi-ring buffers of 50 m, 150 m, and 350 m were established around the areas. Finally, the average Ta data for all regions in each season were summarized.

#### Air quality indicators data

The data for three air quality indicators are sourced from the high-resolution, high-quality near-surface air pollutant dataset for China, published on the National Tibetan Plateau Data Center website by Dr. Jing Wei and Professor Zhanqing Li’s team (ChinaHighAirPollutants, CHAP). The raster data for the three air quality indicators were imported into ArcGIS 10.8 for processing. Using the same processing method as for the air temperature data, the average values of three air quality indicators for each season within the plots and in the external buffer zones were obtained.

### Statistical analysis

This paper primarily utilizes remote sensing data for urban environmental studies. Through the spatial information provided by remote sensing data, certain urban environmental conditions at specific times can be measured, and changes in these conditions over time can be tracked. By integrating data from meteorological stations or ground observation points, the identification of urban environmental data is effectively enhanced (Miller and Small 2003; Musse, Barona, and Santana Rodriguez 2018).

Data analysis was conducted using various statistical charts, including line and bar graphs. Bar graphs can be drawn either horizontally or vertically and include both categorical and quantitative attributes. In this paper, grouped bar charts, stacked bar charts and side-by-side bar charts were used to analyze and illustrate the data. Line graphs, based on the slope of data changes, make it easy to identify time trends and fluctuations, so we created grouped lines to demonstrate the data of the changes. By employing these graphical representation methods, data handling with multiple dimensions and attributes is improved, allowing for a clear expression of the data content (Luo et al. 2019).

### Spatial gradient

Buffer zones can effectively mitigate the spread of pollution. For different pollutants and varying purposes, the width and materials of the buffer zones differ (Cole, Stockan, and Helliwell 2020). Therefore, it is crucial to study the impact of areas with high pollution concentrations on their surrounding environment. Spatial gradient analysis can effectively reveal the changes in pollution concentration within different buffer rings. Thus, a series of concentric buffer rings was generated using the multiple-ring buffer tool in ArcGIS (Liu et al. 2023). Previous studies at the national, city, or even larger global scales were not able to adequately assess risks at the community level. Thus, the study focuses on the buffer zone representing the immediate living community of residents, where circulation can meet most of the daily needs of the residents, and the average walkable neighbourhood area is close to the “5-minute walk” distance of 400 m (Smith et al. 2010; Tenailleau et al. 2015). Therefore, this paper focuses on spatial variations at the neighbourhood scale, generating multiple ring buffers at 50 m, 150 m, and 350 m, respectively, to enhance the understanding of fine spatial scales (Gao et al. 2019; Shairsingh et al. 2018).

## Results

### Yearly temporal evolution of urban heat and pollution

#### Heat

The yearly temporal evolution of temperature for the 12 plots is shown in Supplementary Fig. 2 and Supplementary Fig. 3, which display the yearly temporal evolution of LST and Ta, respectively. Firstly, regarding the internal areas of the plots, over the four years, the least variation in LST data was observed in Plots 1, 6, 8, 9, 10, and 11. This is primarily reflected in the fact that, aside from spring, the fluctuations in LST in other seasons were within 10 °C. For the remaining plots, the fluctuations in LST, excluding spring, were within 15 °C over the four years. At the same time, the Ta remained relatively stable over the four years, with no significant upward or downward trend.

Secondly, for the external areas of the plots, compared to the period before the regeneration, during the final year of the implementation phase of the regeneration, the LST and Ta internal plots are subtracted from the LST and Ta external areas of the plots exhibited significant changes, as detailed in Supplementary Table 2, Supplementary Fig. 4 and Supplementary Fig. 5.

#### Pollution

The yearly temporal evolution of CO, NO_2_, and SO_2_ for the 12 plots is shown in Supplementary Fig. 6, Supplementary Fig. 7, and Supplementary Fig. 8. Firstly, regarding the internal areas of the plots, over the four years, Plot 5 exhibited smoother curves and smaller fluctuations in all three pollutants. The smallest variations in CO concentration were observed in Plots 1, 2, and 3; the smallest variations in NO_2_ data were in Plots 1, 2, 10, and 11; and the smallest variations in SO_2_ concentration data were in Plots 4, 6, 8, and 10.

Secondly, for the external areas of the plots, compared to the period before the regeneration, during the final year of the implementation phase of the regeneration, the CO, NO_2_, and SO_2_ concentrations internal plots are subtracted from the CO, NO_2_, and SO_2_ concentrations external areas of the plots, which exhibited significant changes, are listed in Supplementary Table 3, Supplementary Fig. 9, Supplementary Fig. 10 and Supplementary Fig. 11.

Therefore, from pre-regeneration to the implementation stage of urban regeneration, there is a significant impact on the LST within the plots, whereas there is relatively no major impact on air quality. Moreover, with the implementation of urban regeneration, the temperature difference between the inside and outside of the plots, as well as the differences in CO, NO_2_, and SO_2_ levels, mainly show a decreasing trend.

### Seasonal disparity of urban heat and pollution

#### Heat

**Fig. 2:**
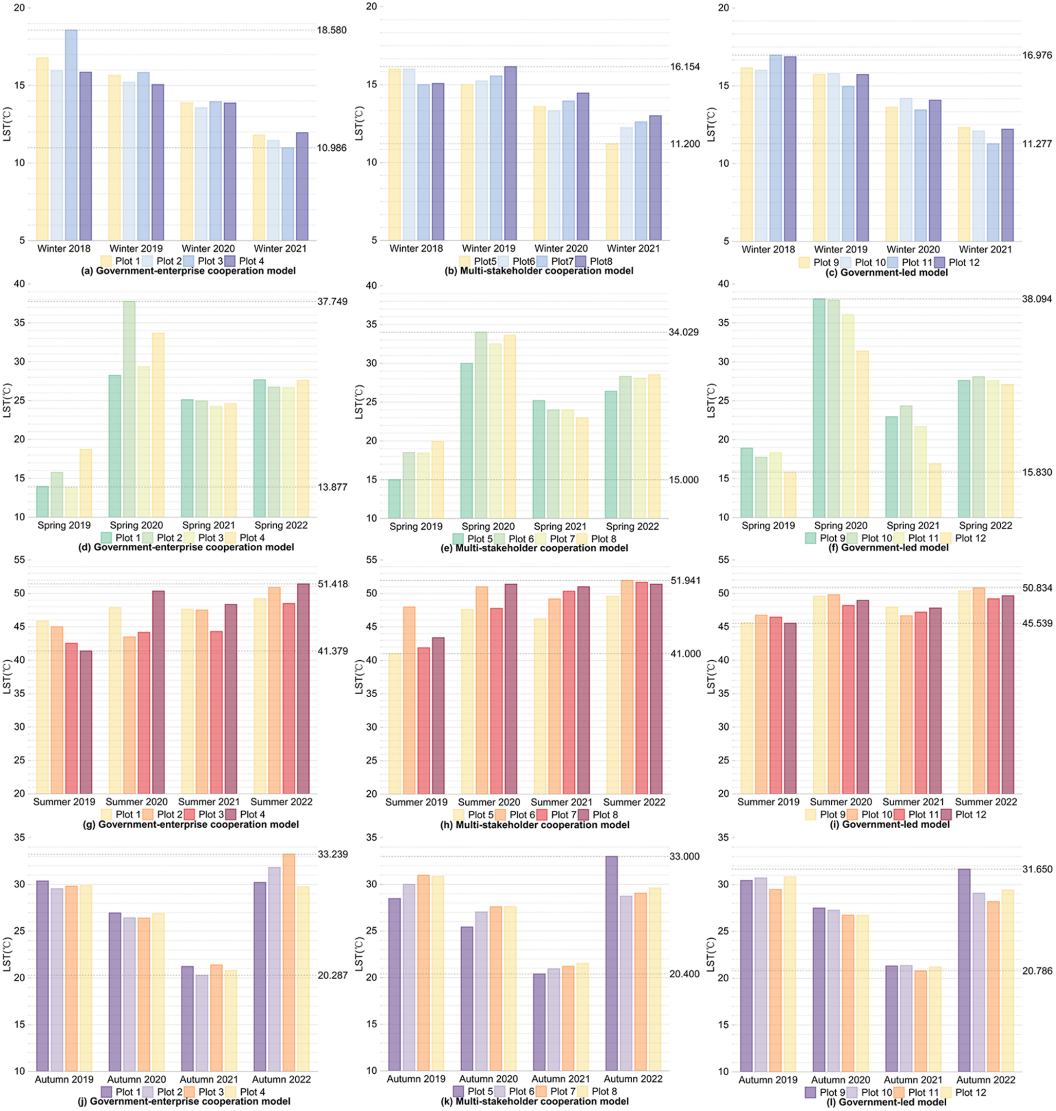
Changes in LST within the plots over the same seasons for four years under different regeneration models

**Fig. 3:**
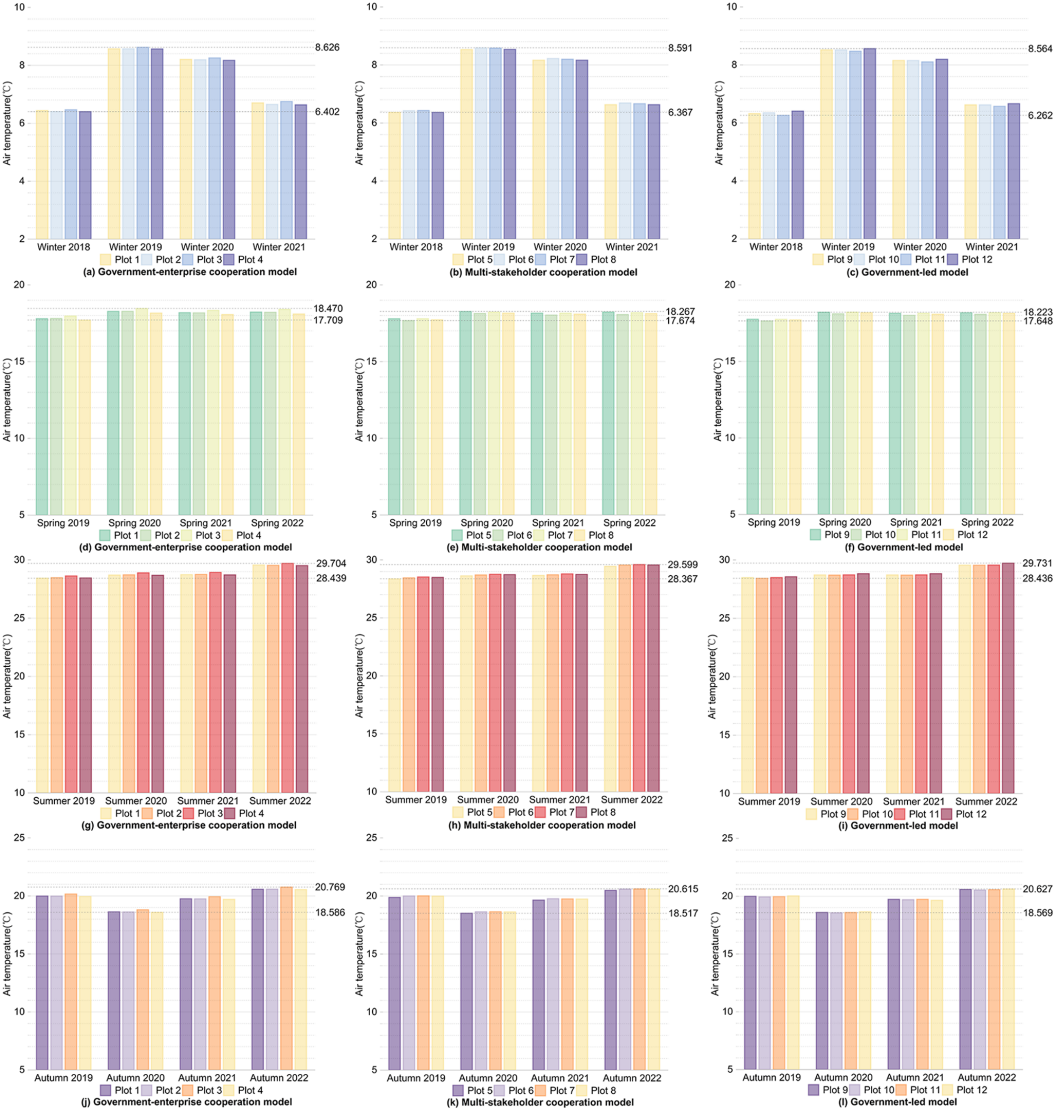
Changes in Ta within the plots over the same seasons for four years under different regeneration models

Firstly, for the internal areas of the plots, as shown in Figs. 2 and 3, during winter, for the 12 plots comparing the winter of 2021 after the regeneration with the winter of 2018 before the regeneration, the overall LST values decreased, with all plots showing a reduction within 10 °C, and the overall Ta values increased, with all plots showing an increase within 1 °C. In spring, for the 12 plots comparing the spring of 2022 after the regeneration with the spring of 2019 before the regeneration, both temperature values overall increased, with the LST of all plots rising within 15 °C and the Ta rising within 1 °C. During summer, comparing the summer of 2022 with the summer of 2019, the LST and Ta changes for the 12 plots over four years generally showed an increasing trend, with the LST of all plots rising within 15 °C and the Ta rising within 2 °C. In autumn, the LST and Ta changes for the 12 plots over four years showed an initial decrease followed by an increase; after four years, there was no significant increase or decrease in LST, while the overall Ta values increased, with all plots showing an increase within 1 °C.

Secondly, for the external areas of the plots, as can be seen from Supplementary Fig. 4 and Supplementary Fig. 5, during winter and spring, the LST in most external areas was lower than that within the plots, but the Ta in most external areas was higher than that within the plots. In summer and most cases, the Ta in the external areas was also higher than within the plots. During autumn, the LST in most external areas was lower than that within the plots. Moreover, over the four years, the changes in external temperatures of all plots generally followed the same trends as those observed internally.

#### Pollution

**Fig. 4:**
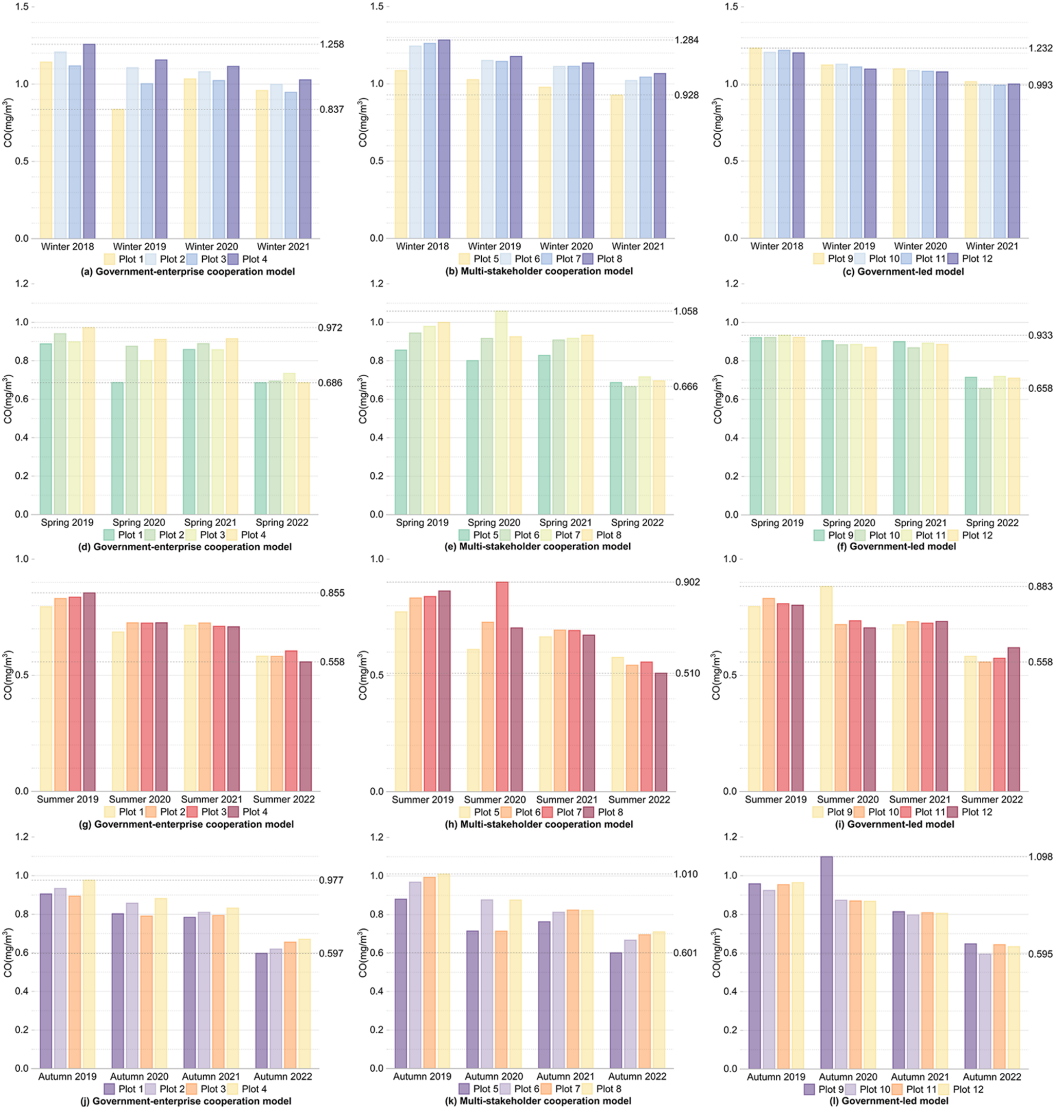
Changes in CO concentration within the plots over the same seasons for four years under different regeneration models

**Fig. 5:**
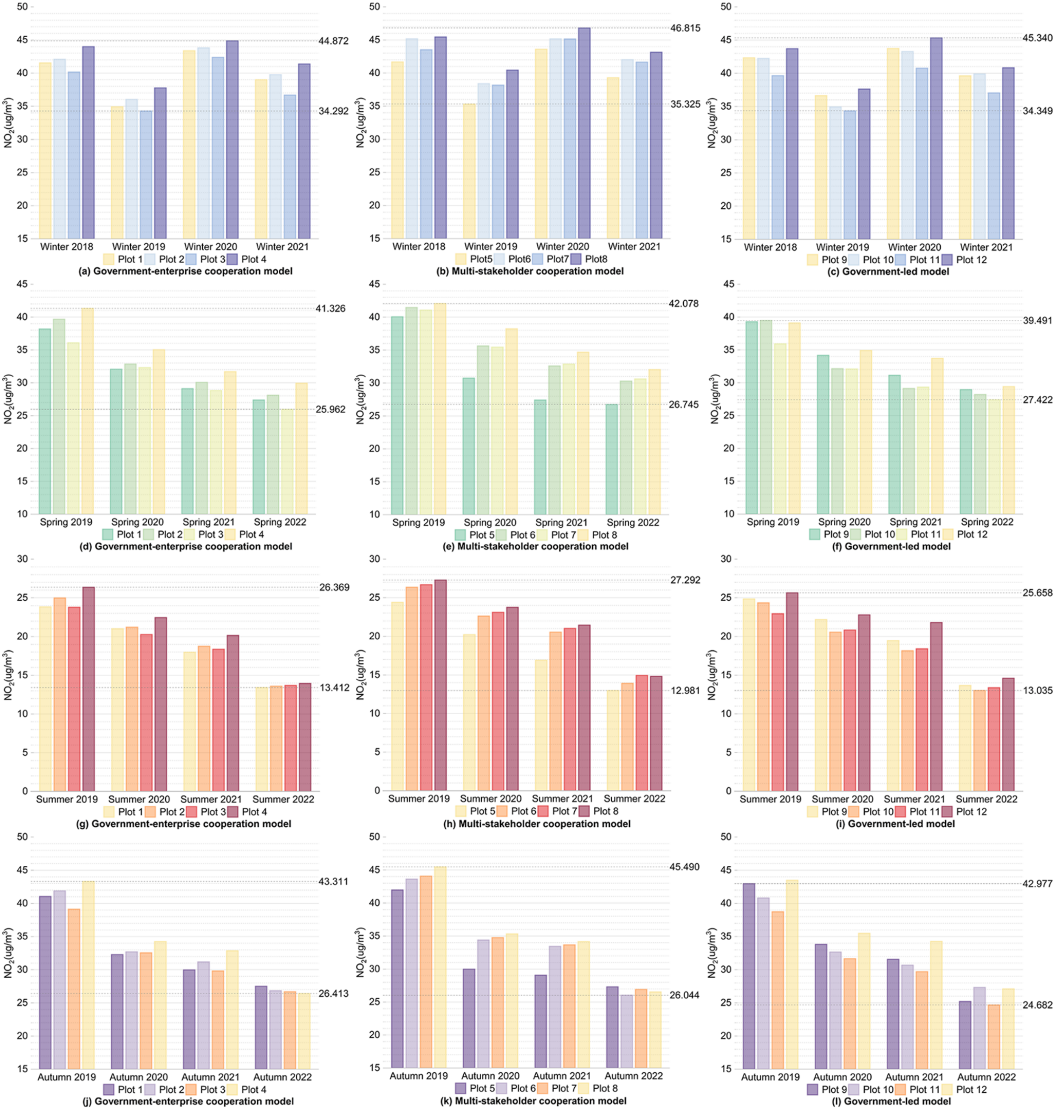
Changes in NO_2_ concentration within the plots over the same seasons for four years under different regeneration models

**Fig. 6:**
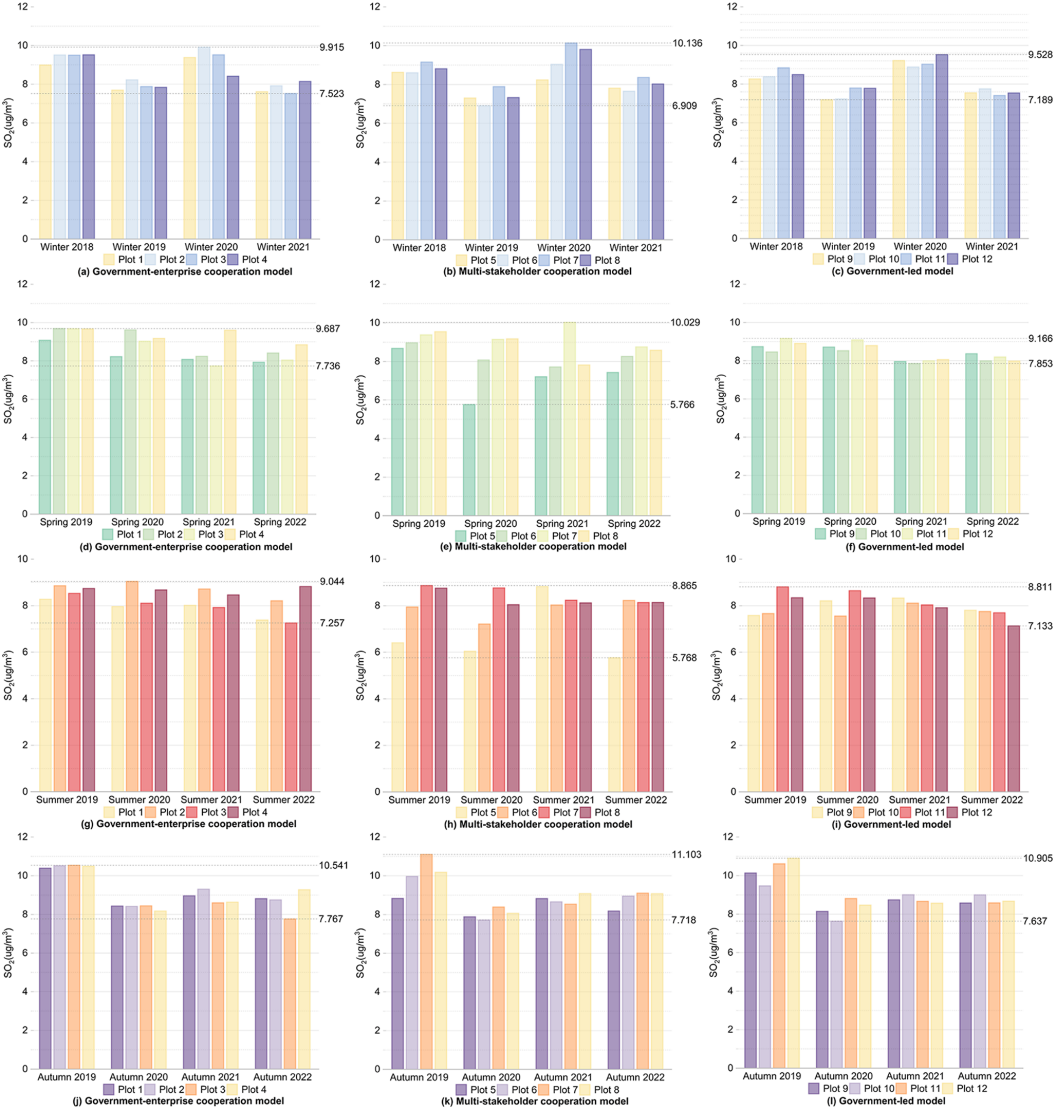
Changes in SO_2_ concentration within the plots over the same seasons for four years under different regeneration models

Firstly, for the internal areas of the plots, as shown in Figs. 4 and 5, and Fig. 6, during winter, except for Plot 1, the CO concentration changes over four years for the other plots generally showed a decreasing trend, with fluctuations within 0.1 mg/m³. The NO_2_ and SO_2_ concentrations comparing the winter of 2021 after the regeneration with the winter of 2018 before the regeneration, the overall values decreased, with all plots showing a reduction in NO_2_ concentration within 5 µg/m³ and a reduction in SO_2_ concentration within 2 µg/m³. In spring and autumn, comparing the spring and autumn of 2022 after the regeneration with the spring and autumn of 2019 before the regeneration, the CO, NO_2_, and SO_2_ concentration changes for the 12 plots over four years generally showed a decreasing trend, the CO concentration decreased within 0.4 mg/m³, the NO_2_ concentration decreased within 20 µg/m³, and the SO_2_ concentration decreased within 3 µg/m³ for all plots. During summer, CO and NO_2_ showed the same changing trends as in spring, with all plots experiencing a decrease in CO concentration within 0.4 mg/m³ and a decrease in NO_2_ concentration within 15 µg/m³. For the pollutant SO_2_, comparing the summer of 2022 with the summer of 2019, Plots 1, 2, 3, 5, 7, 8, 11, and 12 showed an overall decreasing trend in SO_2_ concentration, and the fluctuations in SO_2_ concentration for all plots were within 2 µg/m³.

Secondly, for the external areas of the plots, as can be seen from Supplementary Fig. 9, Supplementary Fig. 10, and Supplementary Fig. 11, in summer, most of the plots had lower external CO concentrations than internal ones. In all four seasons, the NO_2_ concentration in the external areas of most plots was lower than that in the internal areas. Additionally, over the four years, the changes in external air pollutant concentrations for all plots generally followed the same trends as those observed internally.

Therefore, when comparing the last four seasons of the regeneration process with the first four seasons before the regeneration, in winter, the fluctuations in LST, Ta, and the concentrations of the three pollutants within the plots were the smallest. For the external buffer zones of the plots under similar comparison conditions, the Ta was the most affected factor in the external buffer zones throughout the year.

### Seasonal disparity of heat and pollution in different spatial gradients

#### Heat

**Fig. 7:**
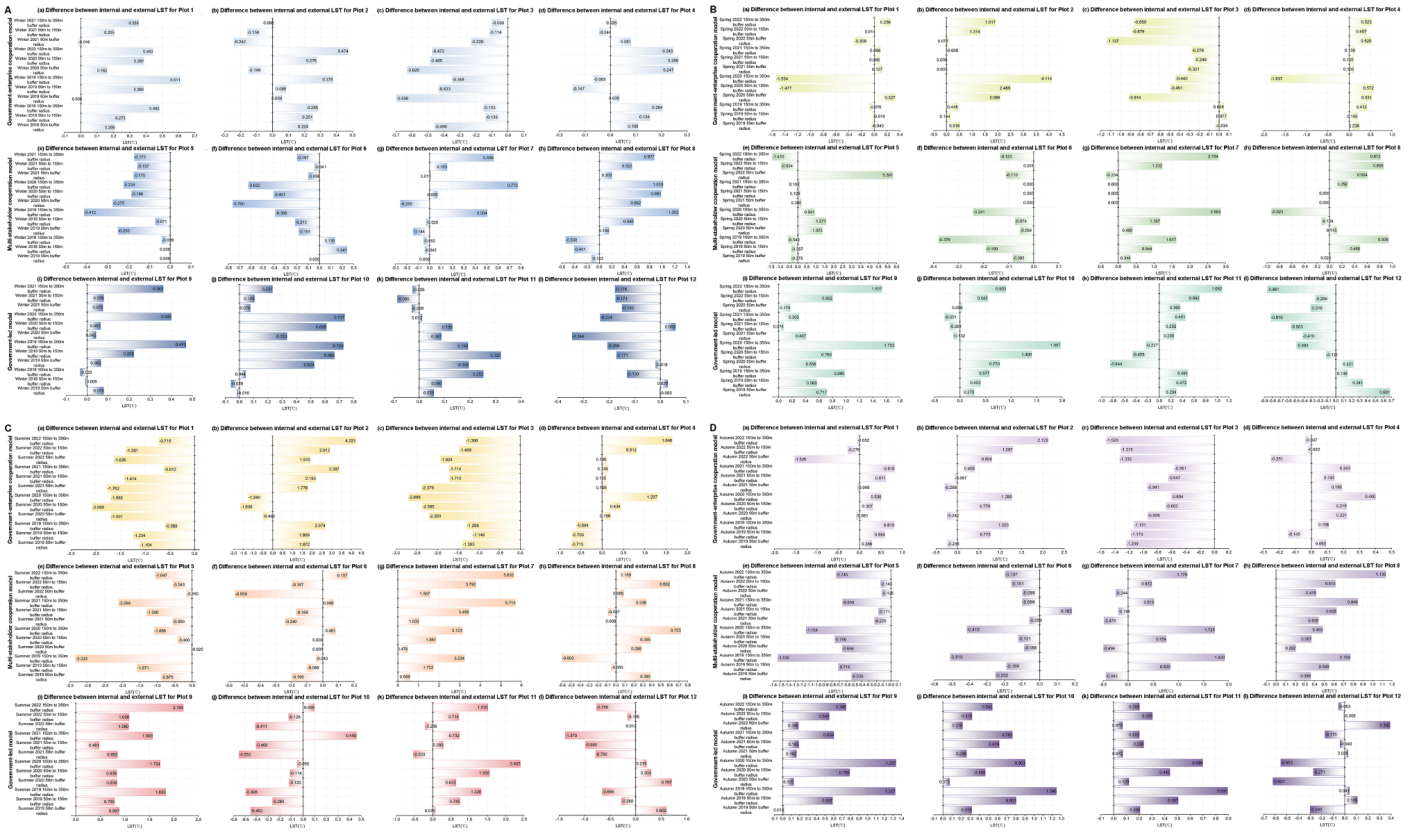
Changes in the difference between internal minus external LST for 12 plots over four years, under four different seasons (winter, spring, summer, and autumn). (**A** - Winter, **B** - Spring, **C** - Summer, **D** - Autumn; in each seasonal graph, colours from light to dark represent three urban regeneration models: government-led, government-enterprise cooperation-led, and multi-stakeholder cooperation-led)

**Fig. 8:**
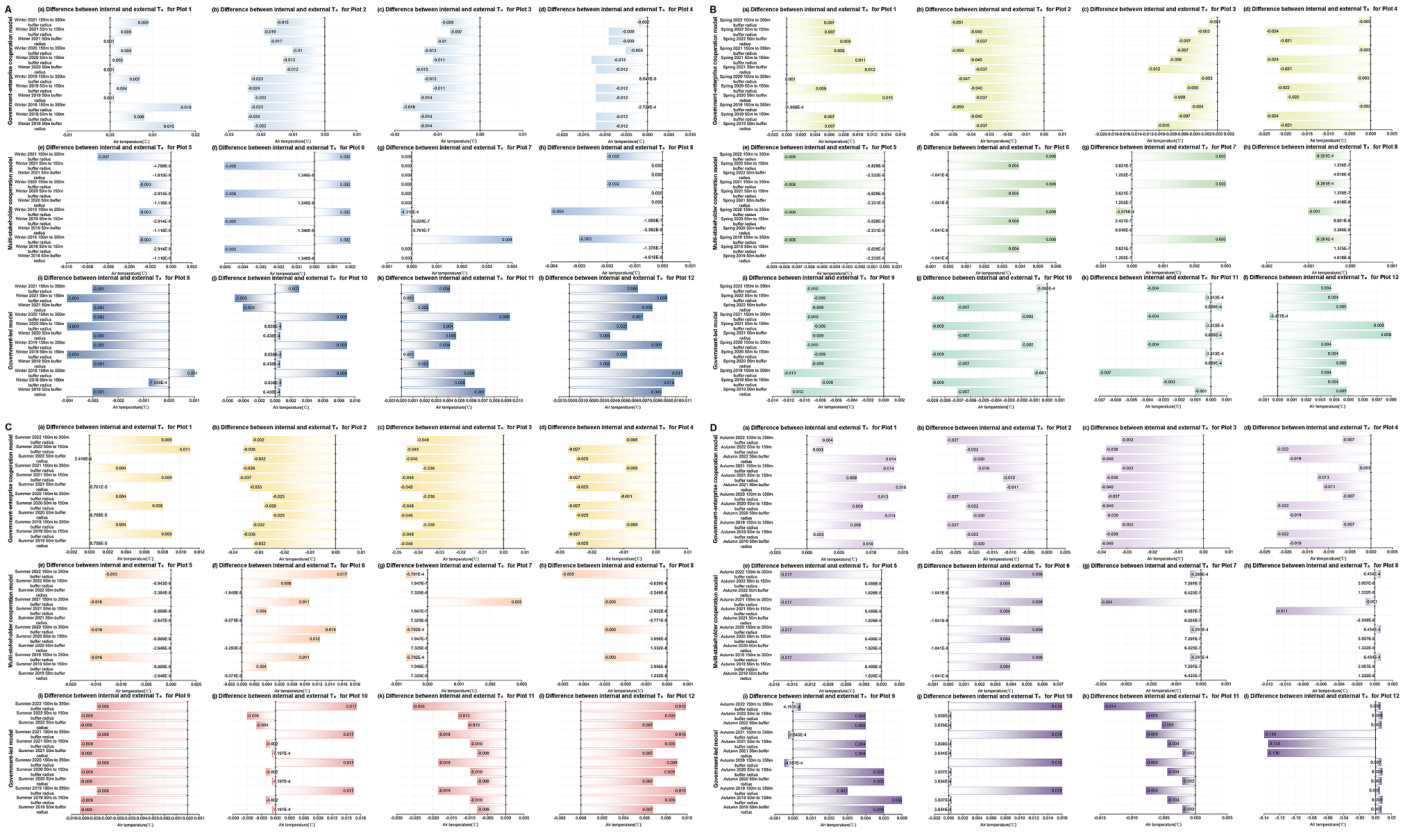
Changes in the difference between internal minus external Ta for 12 plots over four years, under four different seasons (winter, spring, summer, and autumn). (**A** - Winter, **B** - Spring, **C** - Summer, **D** - Autumn; in each seasonal graph, colours from light to dark represent three urban regeneration models: government-led, government-enterprise cooperation-led, and multi-stakeholder cooperation-led)

For the external areas of the plots (as can be seen from Figs. 7 and 8), when comparing the first winter, spring, summer, and autumn before the urban regeneration with the last four seasons after the urban regeneration began, Plots 1, 2, 3, 7, 8, 9, 10, and 11 showed a trend where the LST generally decreased as the distance from the regeneration plot itself increased during most of the last four seasons, the temperature differences within the plots are all within 6℃. Regarding Ta, most of these plots exhibited the same trend, except for Plots 7, 8, 9, and 11, the temperature differences within the plots are all within 0.04℃. The specific characteristics of the external areas of the plots that showed significant changes are detailed in Supplementary Table 4.

#### Pollution

**Fig. 9:**
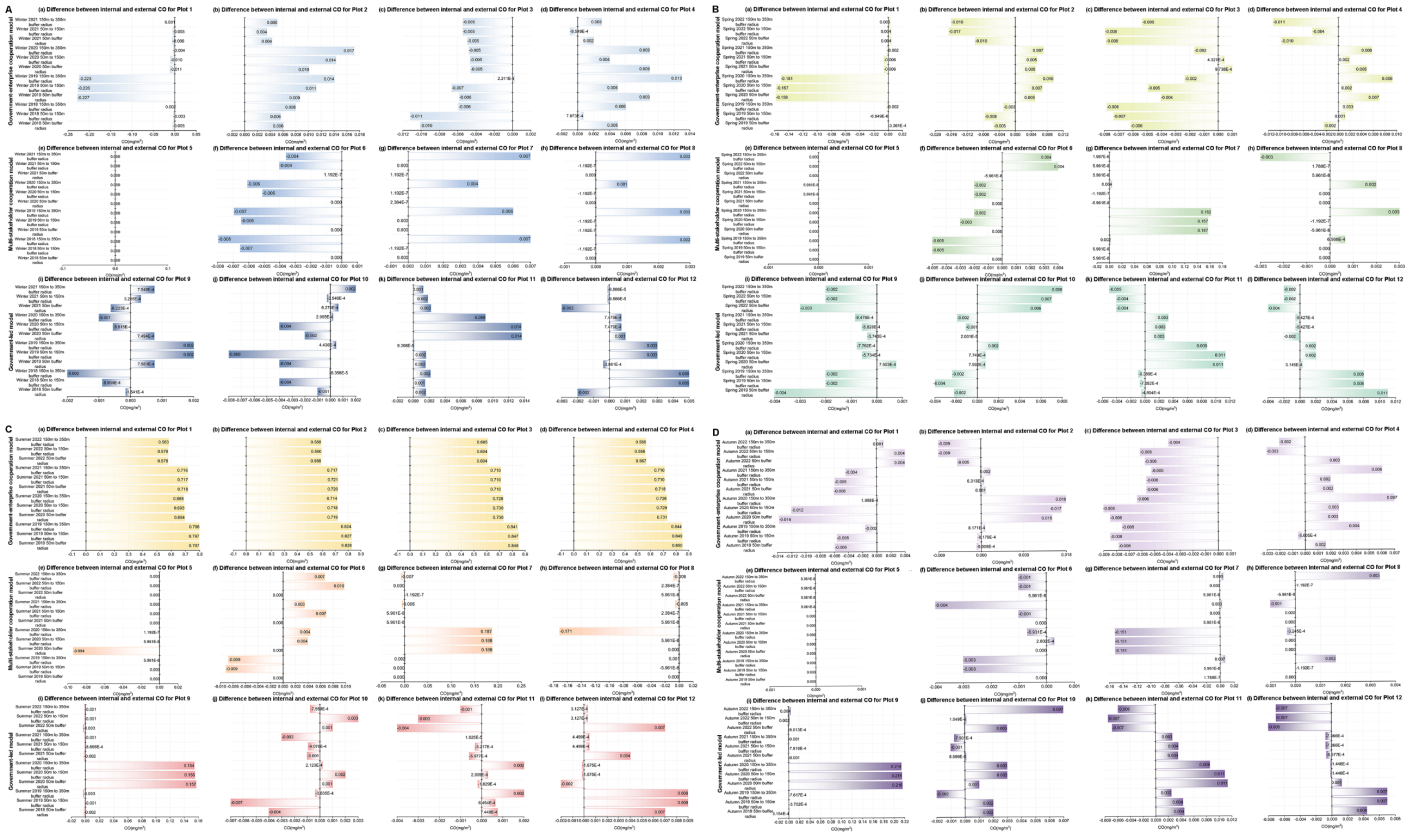
Changes in the difference between internal minus external CO concentrations for 12 plots over four years, under four different seasons (winter, spring, summer, and autumn). (**A** - Winter, **B** - Spring, **C** - Summer, **D** - Autumn; in each seasonal graph, colours from light to dark represent three urban regeneration models: government-led, government-enterprise cooperation-led, and multi-stakeholder cooperation-led.)

**Fig. 10:**
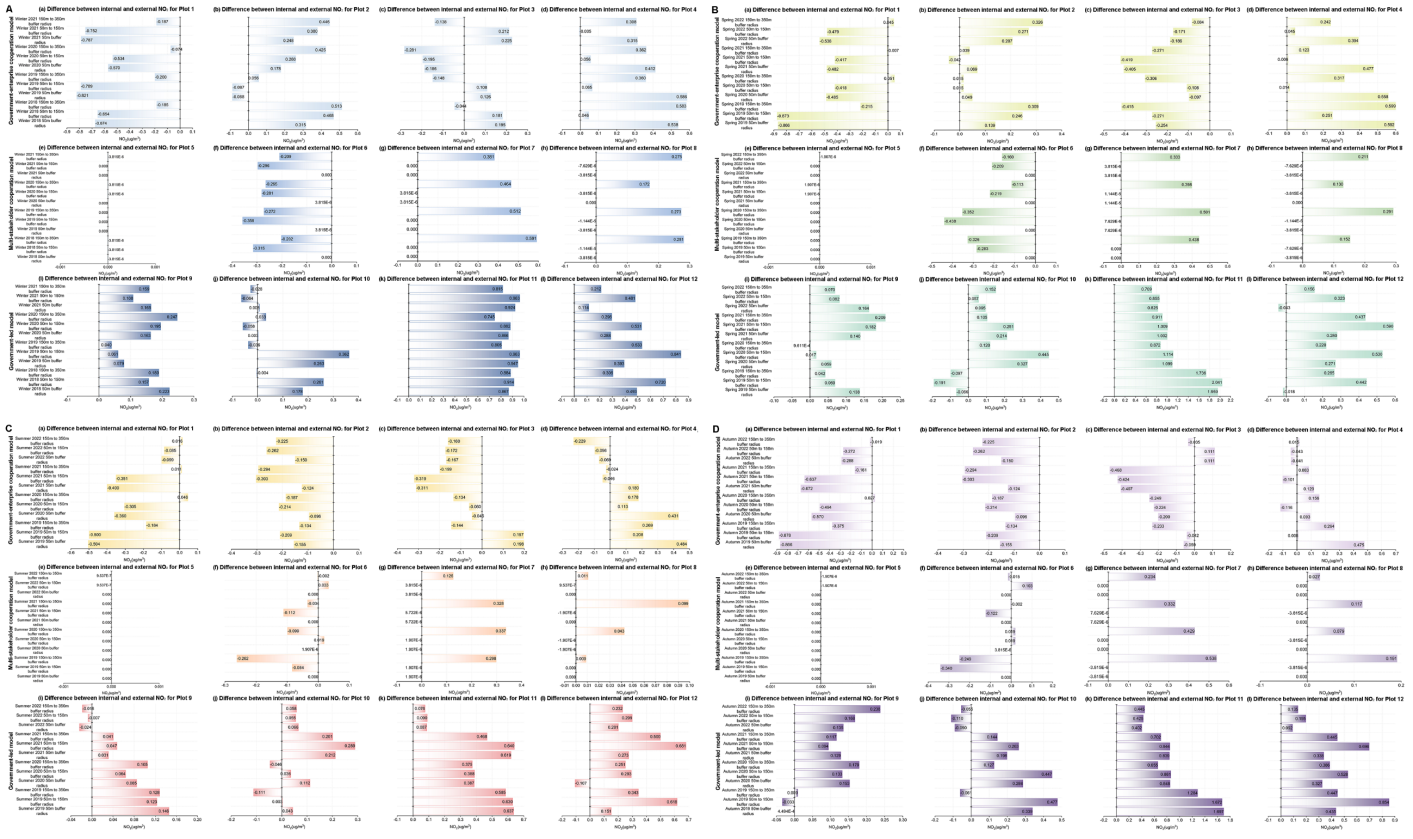
Changes in the difference between internal minus external NO_2_ concentrations for 12 plots over four years, under four different seasons (winter, spring, summer, and autumn). (**A** - Winter, **B** - Spring, **C** - Summer, **D** - Autumn; in each seasonal graph, colours from light to dark represent three urban regeneration models: government-led, government-enterprise cooperation-led, and multi-stakeholder cooperation-led.)

**Fig. 11:**
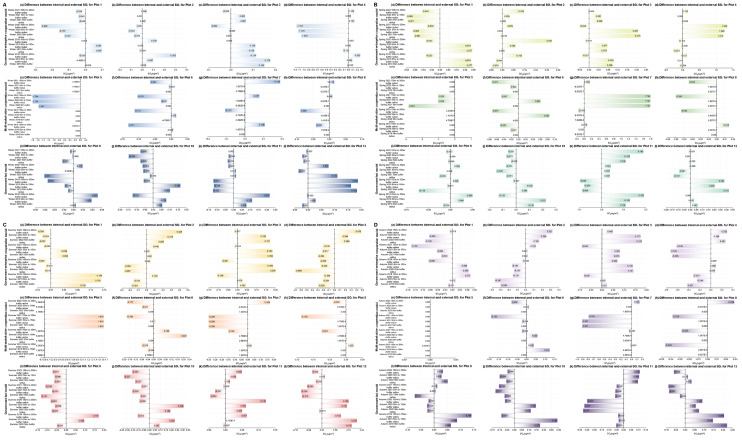
Changes in the difference between internal minus external SO_2_ concentrations for 12 plots over four years, under four different seasons (winter, spring, summer, and autumn). (**A** - Winter, **B** - Spring, **C** - Summer, **D** - Autumn; in each seasonal graph, colours from light to dark represent three urban regeneration models: government-led, government-enterprise cooperation-led, and multi-stakeholder cooperation-led.)

For the external areas of the plots (as can be seen from Figs. 9 and 10, and Fig. 11), when comparing the first winter, spring, summer, and autumn before the urban regeneration with the last four seasons after the urban regeneration began, Plots 2, 7, and 10 showed a trend where the concentrations of the three pollutants generally decreased as the distance from the regeneration plot itself increased during most of the last four seasons. The concentration differences of the three pollutants CO, NO_2_, and SO_2_ outside the plots compared to inside are all within 0.9 mg/m³, 0.9 µg/m³, and 1.5 µg/m³, respectively. The specific characteristics of the external areas of the plots that showed significant changes are detailed in Supplementary Table 5.

Therefore, during the implementation phase of urban regeneration, LST mostly shows a trend of decreasing as the distance from the urban regeneration plots increases. However, there are no significant trends in Ta or concentrations of CO, NO_2_, and SO_2_.

### Multi-stakeholder effects of heat and pollution evolution

#### Heat

Firstly, as can be seen from Figs. 2 and 3, and shown in Supplementary Tables 6 and Supplementary Table 7, for the internal areas of the plots, the government-enterprise cooperation-led urban regeneration model showed the greatest overall fluctuation in LST over four years. Furthermore, under this model, in the spring of 2022, after the regeneration began, the overall LST of the four plots compared to all models in the same season were lower, all below 28 °C, and generally about 0.5 °C lower than other plots. For the urban regeneration model led by multi-stakeholder cooperation, the overall Ta in the summer and autumn of 2022 compared to all models in the same season was lower, generally about 0.1 °C below those of other plots. For the government-led urban regeneration model, the overall fluctuation in LST over four years was the smallest. Under this model, the LST in the winter of 2021, summer of 2022, and autumn of 2022 compared to all models in the same season were lower, with winter temperatures generally at or below 12 °C, summer temperatures below 51 °C, and autumn temperatures below 30 °C, all approximately 1 °C lower than other plots. The Ta in the winter of 2021 and spring of 2022 compared to all models in the same season were also lower, about 0.1 °C below other plots.

Secondly, for the external areas of the plots, as can be seen from Figs. 7 and 8, Supplementary Tables 6 and Supplementary Table 7, in the government-enterprise cooperation-led urban regeneration model, during the last four seasons after the regeneration began, there was an overall trend of lower LST with increasing distance from the centre of the regeneration plot. Only Plots 3 and 4 showed a trend of lower Ta with increasing distance from the centre. Under the regeneration model led by multi-stakeholder cooperation, there was no clear trend in external LST; however, for Ta in the last four seasons after the regeneration, Plots 5 and 8 exhibited higher Ta with increasing distance from the centre during the last winter, spring, and summer, whereas Plot 6 showed a trend of lower Ta with increasing distance from the centre. For the government-led urban regeneration model, in the last four seasons after the regeneration, all areas except for Plot 12 generally showed a trend of lower LST with increasing distance from the centre. Meanwhile, only Plot 10 showed a trend of lower Ta with an increasing distance from the centre. Additionally, for the different stakeholder-led urban regeneration models, the variations in LST and Ta within and outside the plots are shown in Supplementary Tables 6 and Supplementary Table 7.

#### Pollution

Firstly, for the internal areas of the plots, as shown in Figs. 4 and 5, and Fig. 6, and shown in Supplementary Table 8, Supplementary Tables 9, and Supplementary Table 10, the government-enterprise cooperation-led urban regeneration model exhibited the smallest overall fluctuations in CO and NO_2_ concentrations over four years, while the fluctuations in SO_2_ concentration were relatively larger. Furthermore, under this model, the overall CO concentration during the winter of 2021, after the regeneration began, was lower than that of other models in the same season, generally below 1 mg/m³, about 0.05 mg/m³ lower than other plots. At the same time, the overall NO_2_ concentration in the spring and summer of 2022 was lower than that of other models in the same season, with spring levels generally below 28 µg/m³ and summer levels below 14 µg/m³, approximately 1 µg/m³ lower than other plots.

For the urban regeneration model led by multi-stakeholder cooperation, the overall fluctuation in NO_2_ concentration over four years was the largest, while the fluctuation in SO_2_ concentration was the smallest. In the spring and summer of 2022, the overall CO concentration was lower compared to other models in the same seasons, with spring concentrations generally below 0.7 mg/m³ and summer concentrations below 0.6 mg/m³, both approximately 0.05 mg/m³ lower than other plots.

For the government-led urban regeneration model, the overall fluctuations in CO and SO_2_ concentrations over four years were the largest, while the fluctuation in NO_2_ concentration was relatively smaller. In the plots under this model, after the regeneration began, the overall CO, NO_2_, and SO_2_ concentrations in the autumn of 2022 were lower compared to other models in the same season, with CO concentrations generally around 0.65 mg/m³, NO_2_ concentrations around 26 µg/m³, and SO_2_ concentrations around 8.5 µg/m³. The CO concentrations were about 0.1 mg/m³ lower, NO_2_ concentrations about 1 µg/m³ lower, and SO_2_ concentrations about 0.5 µg/m³ lower than those of other plots. In the winter of 2021, following the start of the regeneration, the overall NO_2_ and SO_2_ concentrations were also lower compared to other models in the same season, with NO_2_ concentrations generally below 40 µg/m³ and SO_2_ concentrations below 7.5 µg/m³, both approximately 1 µg/m³ and 0.3 µg/m³ lower, respectively, than other plots. Additionally, the SO_2_ concentrations in the spring and summer of 2022, after the regeneration, were lower, with spring concentrations around 8 µg/m³ and summer concentrations around 7.5 µg/m³, both about 0.5 µg/m³ lower than other plots.

Secondly, as can be seen from Figs. 9, 10 and 11, Supplementary Table 8, Supplementary Tables 9, and Supplementary Table 10, for the external areas of the plots, in the government-enterprise cooperation-led urban regeneration model, during the last four seasons after the regeneration began, Plots 2 and 3 generally showed a trend of lower CO concentrations with increasing distance from the centre of the regeneration plot. Only Plots 1 and 2 exhibited a trend of lower NO_2_ concentrations with increasing distance from the centre. Additionally, except for Plot 3, there was a general trend of lower SO_2_ concentrations with increasing distance from the centre during the last four seasons after the regeneration.

For the regeneration model led by multi-stakeholder cooperation, during the last four seasons after the regeneration began, Plot 7 generally showed a trend of lower CO, NO_2_, and SO_2_ concentrations with increasing distance from the centre. Plot 8, influenced by NO_2_ concentration, also showed the same overall trend.

For the government-led regeneration model, during the last four seasons after the regeneration began, there was generally a trend of lower CO and SO_2_ concentrations with increasing distance from the centre, while there was no clear trend for NO_2_ concentration. Meanwhile, for the different stakeholder-led urban regeneration models, the variations in CO, NO_2_, and SO_2_ concentrations within and outside the plots are shown in Supplementary Table 8, Supplementary Tables 9, and Supplementary Table 10.

Therefore, in the government-enterprise cooperation-led urban regeneration model, compared to other models, there is a larger fluctuation in LST and SO_2_ concentration within the plots, while fluctuations in CO and NO_2_ concentrations are smaller. For the external areas of the plots, there is an overall trend of lower LST, Ta, and CO concentration with increasing distance from the plot in the last four seasons. In the urban regeneration model led by multi-stakeholder cooperation, compared to other models, there is a larger fluctuation in NO_2_ concentration and a smaller fluctuation in SO_2_ concentration within the plots. For the external areas of the plots, there is an overall trend of lower NO_2_ concentration with increasing distance from the plot in the last four seasons. In the government-led urban regeneration model, compared to other models, there is a smaller fluctuation in LST and NO_2_, but a larger fluctuation in CO and SO_2_ concentrations within the plots. For the external areas of the plots, there is an overall trend of lower LST, CO, and SO2 concentrations with increasing distance from the plot in the last four seasons.

## Discussion

### Policy implications

Urban regeneration plays a significant role in research that addresses various urban issues arising from urbanization. To truly achieve sustainable development in urban regeneration, it is necessary to comprehensively consider the different stages of urban regeneration and the complex factors involved in regeneration models led by different stakeholders. This study examines the impact of urban regeneration implementation phases on urban heat and pollution, as well as spatial gradient analysis, under government-enterprise cooperation-led, multi-stakeholder cooperation-led, and government-led urban regeneration models. It aims to better balance the relationship between urban regeneration and the urban environment and to provide more comprehensive assistance for future urban regeneration in terms of stakeholder selection, planning recommendations, and policy-making (S. Zheng, Chen, and Liu 2023).

Previous studies have shown that, since the majority of urban regeneration projects in China are government-led, the government plays a significant role in the decision-making process for urban regeneration (Zhuang et al. 2019). For government-led, top-down urban regeneration, because the government can wield more power and financial resources, it ensures that projects are completed more efficiently and smoothly, avoiding the conflicts among different stakeholders that can arise from a bottom-up urban regeneration approach (Wang et al. 2022).

The research findings of this paper indicate that, after the commencement of urban regeneration, the government-enterprise cooperation-led model has a relatively smaller impact on internal plot urban pollution. The multi-stakeholder cooperation-led urban regeneration model shows a consistent level of impact on both urban heat and pollution within the plots. The government-led urban regeneration model has a relatively smaller effect on urban heat. Additionally, when comparing the last year after the start of regeneration with the first year before the start, the fluctuations in urban heat and pollution within the plots during winter across all four seasons were the smallest. This is consistent with existing research findings that suggest extreme heat island effects are more likely to be induced in hot weather compared to cold weather (Yao et al. 2020).

Therefore, these results confirm that urban regeneration models led by government involvement are more likely to reduce the impact of various sectors’ activities on urban heat and pollution. Moreover, during the winter season across all four seasons, the fluctuations in urban heat and pollution within the plots as a result of urban regeneration implementation are the smallest. Hence, when selecting an urban regeneration model, it is appropriate to increase the weight given to government participation in urban regeneration projects. By managing different stakeholders through government departments, their relationships can be coordinated, avoiding conflicts of interest that could lead to the failure of regeneration projects (Wang et al. 2017). Government departments can promote coordinated development among stakeholders through fair supervision and need to consider the long-term social public interest rather than focusing solely on immediate benefits. Ultimately, they should define the boundaries of participation and scope of responsibilities for all parties, ensuring the coordinated development of multiple stakeholders (Wang et al. 2021a, b). The government especially needs to pay attention to the rights and interests of residents, taking into account the impact of environmental pollution on the population (Yuan 2024). Due to seasonal variations, urban activities that are likely to have a significant impact on urban heat during the urban regeneration process can be implemented in winter, which may reduce their impact on the urban heat environment compared to other seasons. Therefore, urban planning and policy-making can improve the urban heat and pollution effects brought about by urban regeneration, leading to a more livable urban environment, through the selection and responsibility allocation of different stakeholders, as well as the implementation of projects in different seasons.

### Limitations and perspectives

This study has certain limitations. Firstly, the selection of the study area is limited, with a restricted number of available research areas based on specific times and regeneration models, as well as being influenced by the diversity of urban regeneration scales. This results in some plots within the study area being relatively small, and the precision of the data obtained for the regeneration projects is limited. Therefore, the findings may not be representative of all scales of urban regeneration projects, and the results should be applied to other situations with caution.

Secondly, the limitation of data acquisition, due to the limited spatiotemporal resolution of the obtained data, results in a time coverage that does not encompass longer periods of the urban regeneration implementation phase and leads to insufficient data precision. It is not possible to comprehensively observe the impact of demolition and reconstruction on the environment during the entire implementation phase of urban regeneration, nor is it possible to determine how the environmental impact changes as the implementation of urban regeneration comes to an end. This may affect the stability of the results.

Therefore, to address these limitations, future research should also consider a broader range of urban regeneration cases with different scales to investigate the impact of various stakeholder involvements on urban heat and pollution, and conduct a more comprehensive assessment by taking into account different stages of urban regeneration. Additionally, efforts should be made to utilize data sources with higher spatiotemporal resolution and varying temporal resolutions, providing greater data accuracy and a more complete timeline for exploring each phase of urban regeneration.

## Conclusions

In rapid urbanization, a series of environmental pollution issues such as urban heat islands and air pollution have been triggered. How to avoid exacerbating these environmental problems through urban regeneration and truly achieve the sustainability of urban regeneration has become an important issue in the current urban development process. Facing complex urban regeneration projects, it has become increasingly important to study the issues of urban heat and pollution influenced by different stakeholders during the implementation phase of urban regeneration. The following conclusions can be drawn from the research:

1. Comparing the annual changes during the regeneration process, from the pre - regeneration stage to the implementation phase of urban regeneration, the LST within the plots is significantly affected, showing an overall upward trend. In contrast, air quality is relatively less impacted, exhibiting a general downward trend.
2. Comparing the first year before the start of regeneration with the last year of the implementation phase as investigated in this study, during winter, the impact on urban heat and pollution within the plots is the smallest. Additionally, among the four seasons, the most affected parameter in the external buffer zones is air temperature, which, during winter, spring, and summer, is generally higher in the external buffer zones than within the plots.
3. The study revealed that the government-enterprise cooperation-led regeneration model has a relatively smaller impact on internal plot urban pollution. In this model, the temperature of the external buffer zone is less affected by the regeneration. The government-led urban regeneration model has a relatively smaller effect on internal plot urban heat. In this model, the LST, CO, and SO2 concentrations in the external buffer zone are less affected by the regeneration.

## Electronic supplementary material

Below is the link to the electronic supplementary material.

Supplementary Material 1

## Data Availability

The data that supports the findings of this study are available from the corresponding author, upon reasonable request.
