# Supplementary material for: Multi-stakeholder perspectives on urban regeneration: a spatial gradient analysis of heat and pollution effects
Source: Int J Biometeorol. 2025 Mar 24;69(6):1325–45. doi: 10.1007/s00484-025-02894-8 (PMC12141161; doi:10.1007/s00484-025-02894-8)
Supplement: Supplementary file 1 — Supplementary Material 1 [file 484_2025_2894_MOESM1_ESM.docx]

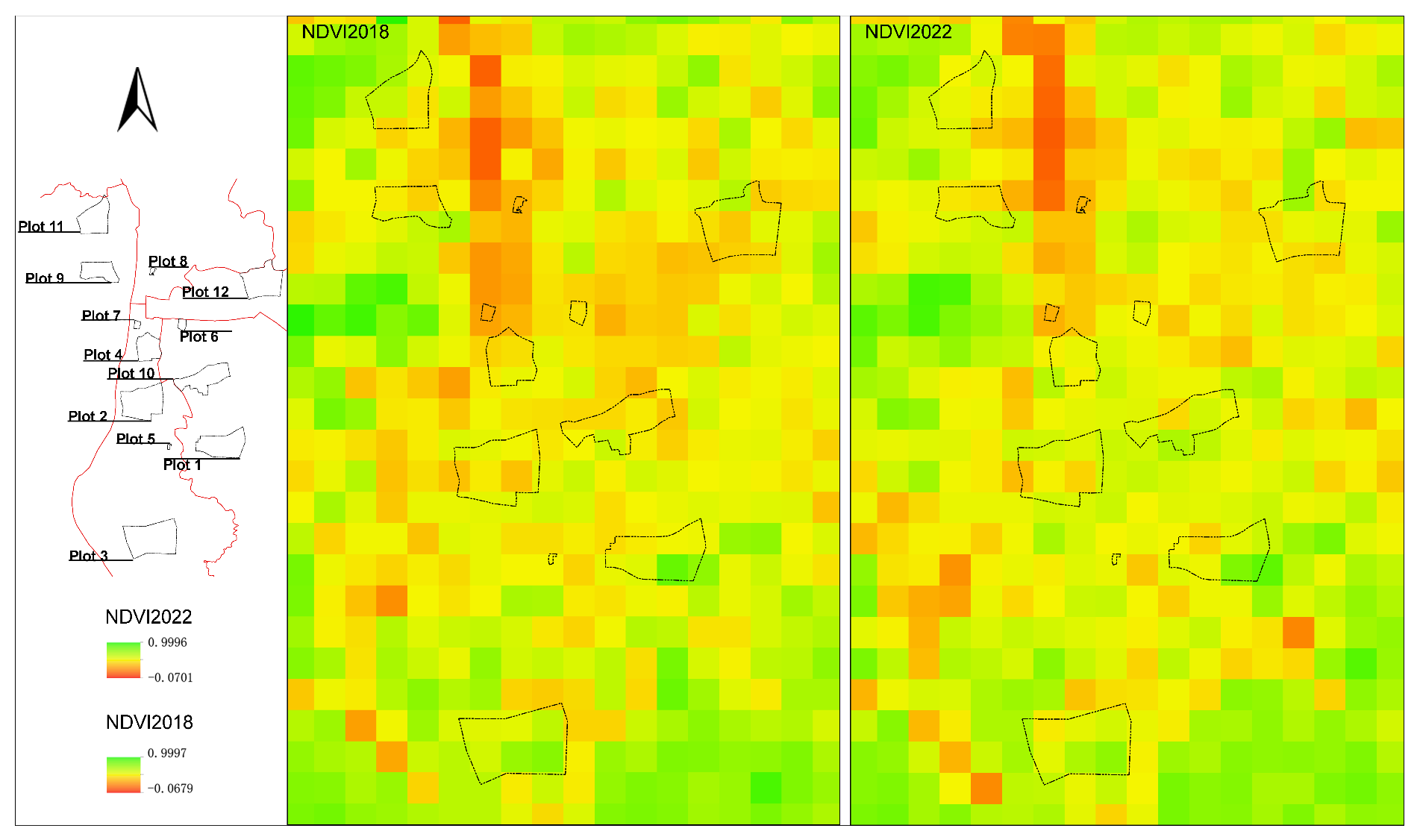


Supplementary Figure 1. The normalized difference vegetation index of 12 plots for the years 2018 and 2022.


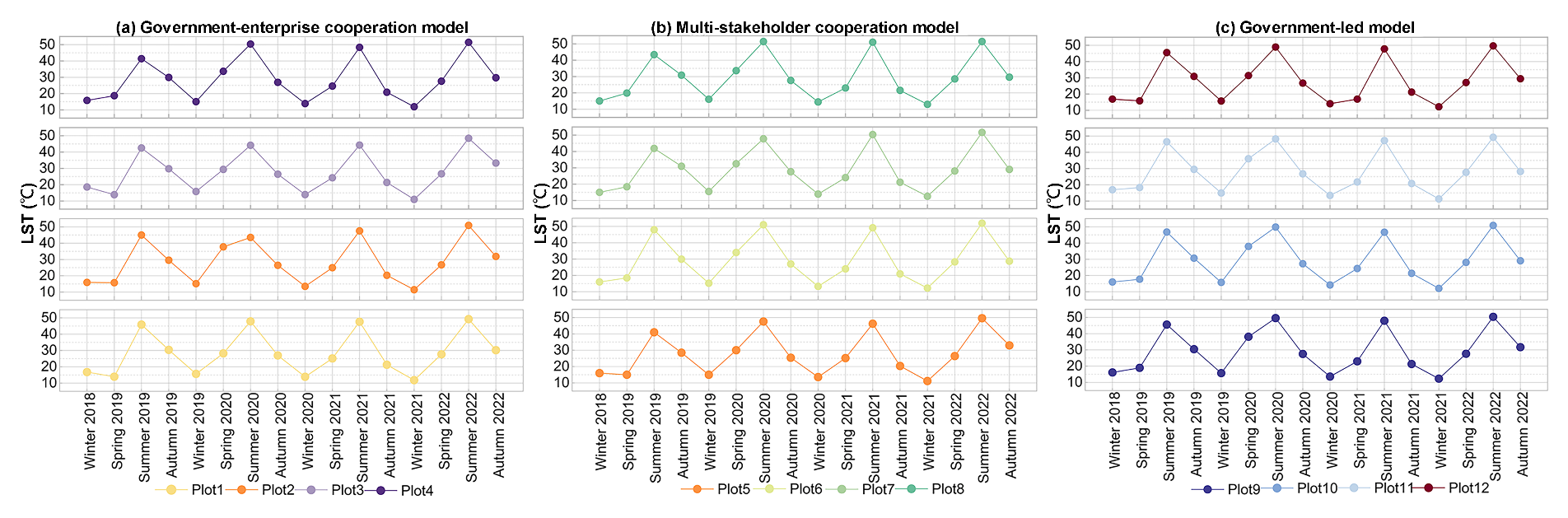


Supplementary Figure 2. Annual temporal evolution of LST within the plots under the same regeneration model.


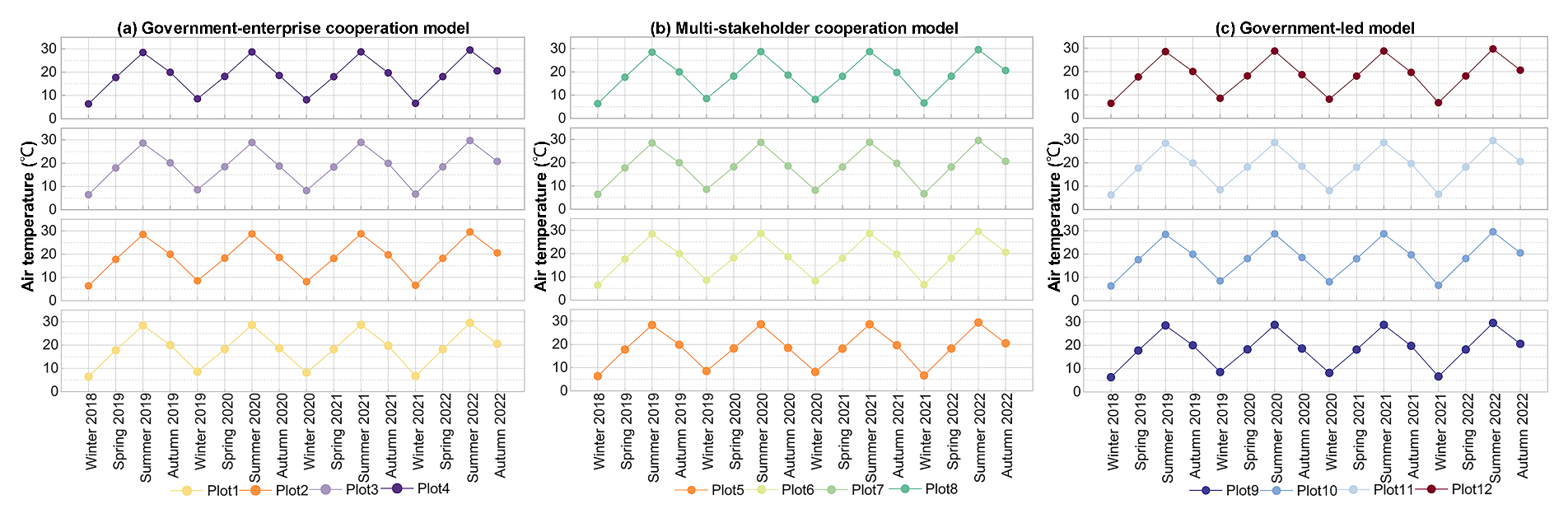


Supplementary Figure 3. Annual temporal evolution of Ta within the plots under the same regeneration model.


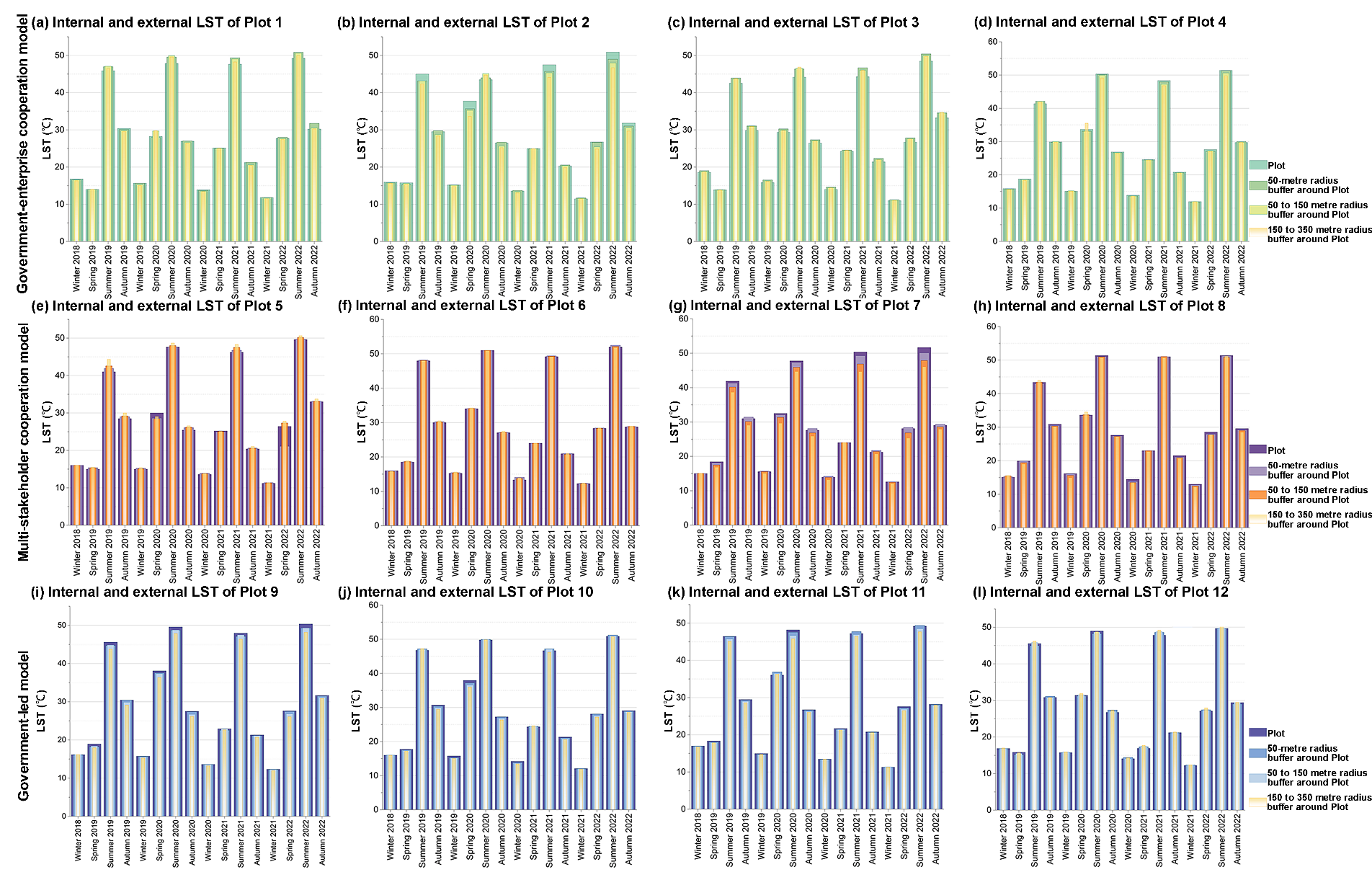


Supplementary Figure 4. LST values within the plot and in three external buffer zones over a four-year period for the same plot.


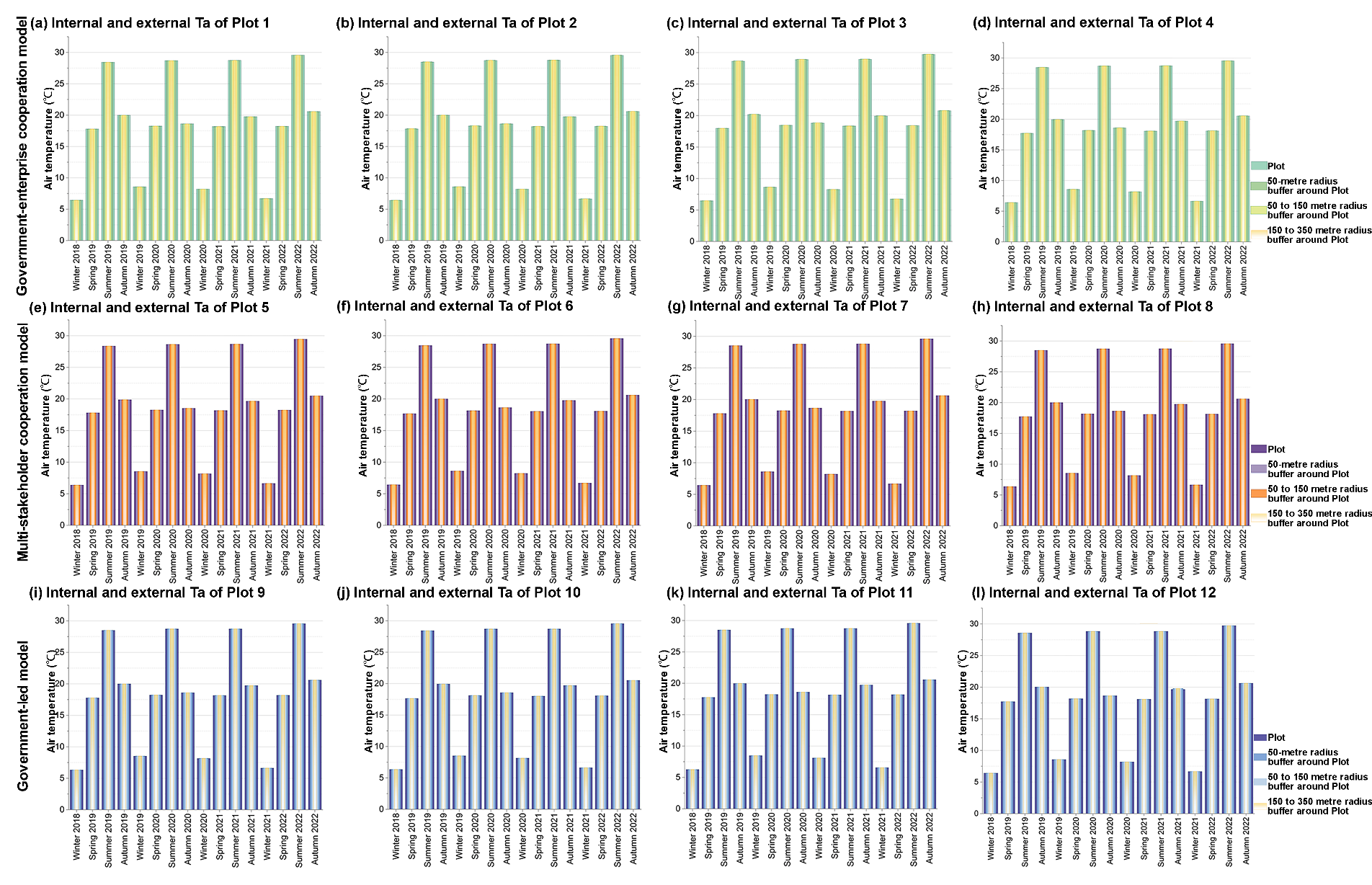


Supplementary Figure 5. Ta values within the plot and in three external buffer zones over a four-year period for the same plot.


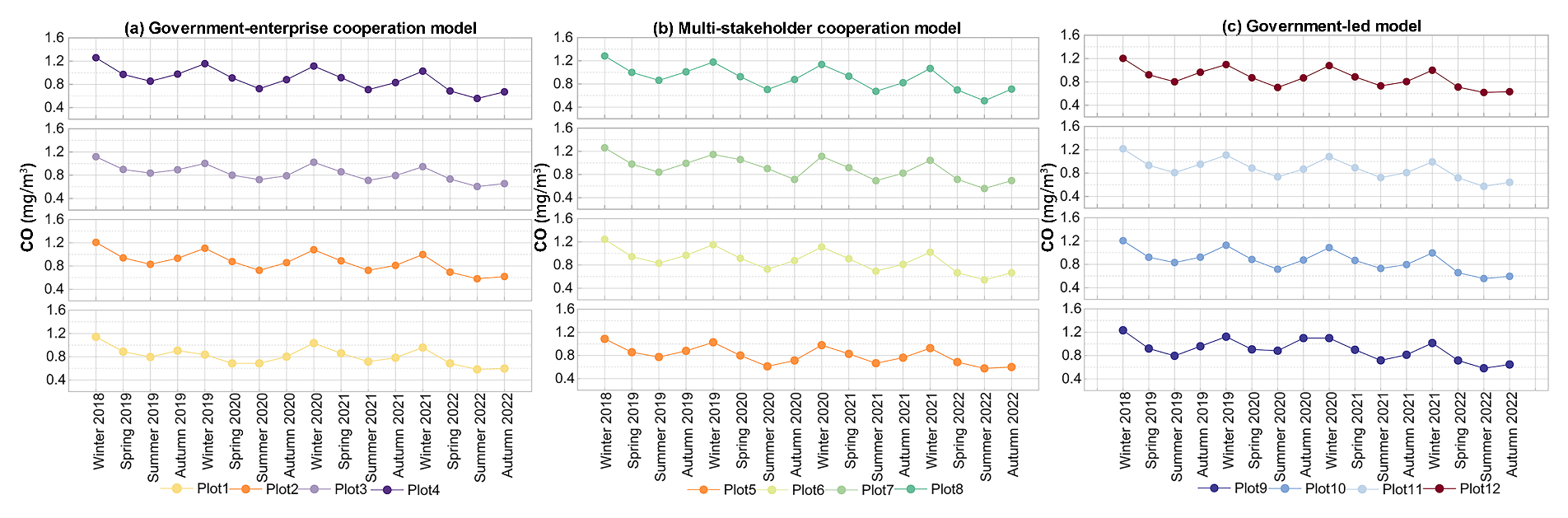


Supplementary Figure 6. Annual temporal evolution of CO concentration within the plots under the same regeneration model.


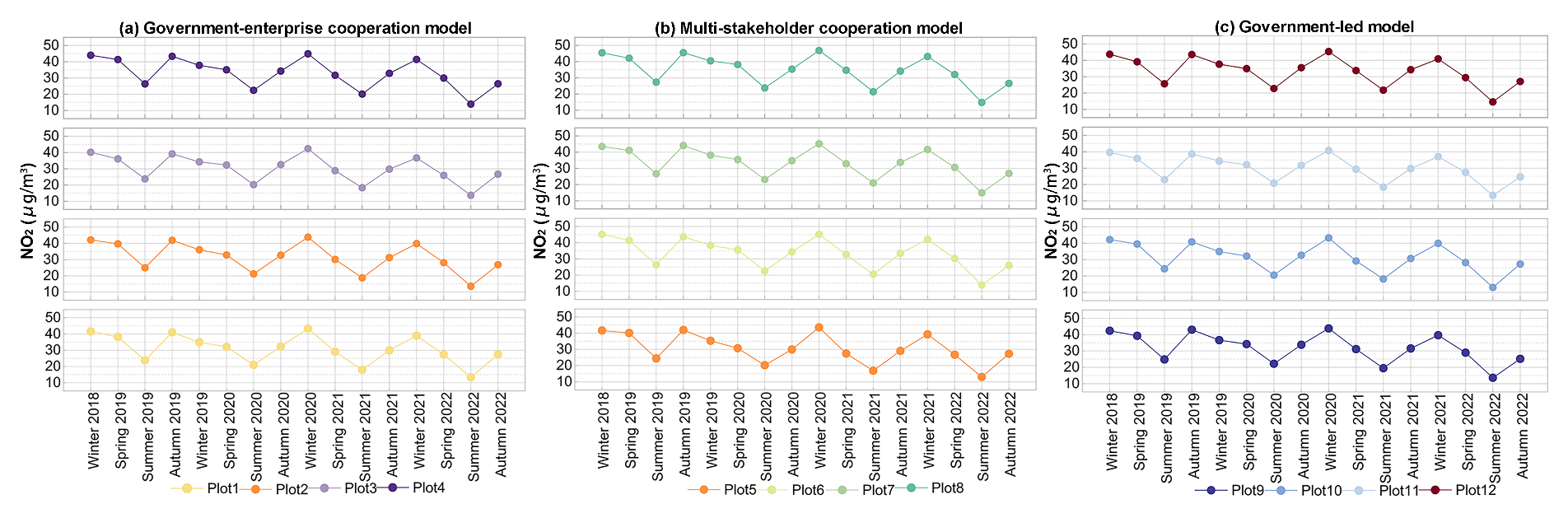


Supplementary Figure 7. Annual temporal evolution of NO_2_ concentration within the plots under the same regeneration model.


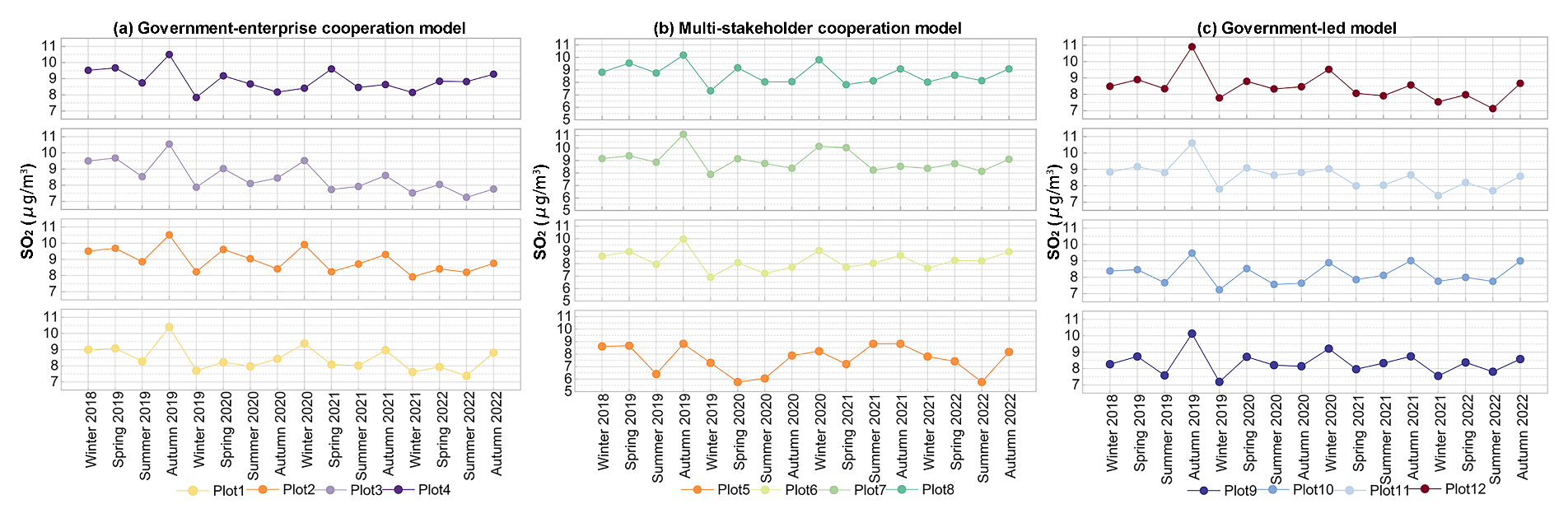


Supplementary Figure 8. Annual temporal evolution of SO_2_ concentration within the plots under the same regeneration model.


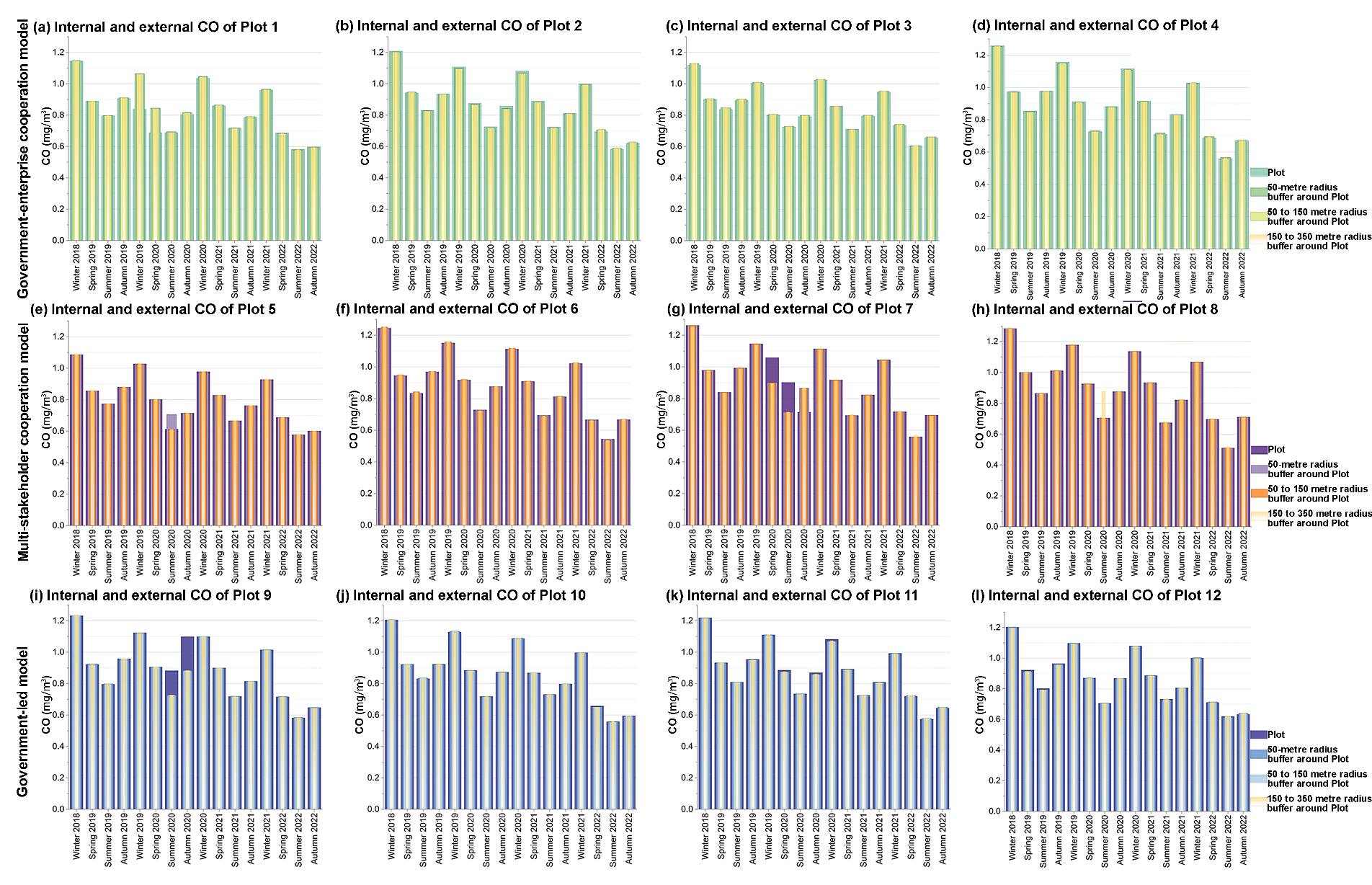


Supplementary Figure 9. CO concentration values within the plot and in three external buffer zones over a four-year period for the same plot.


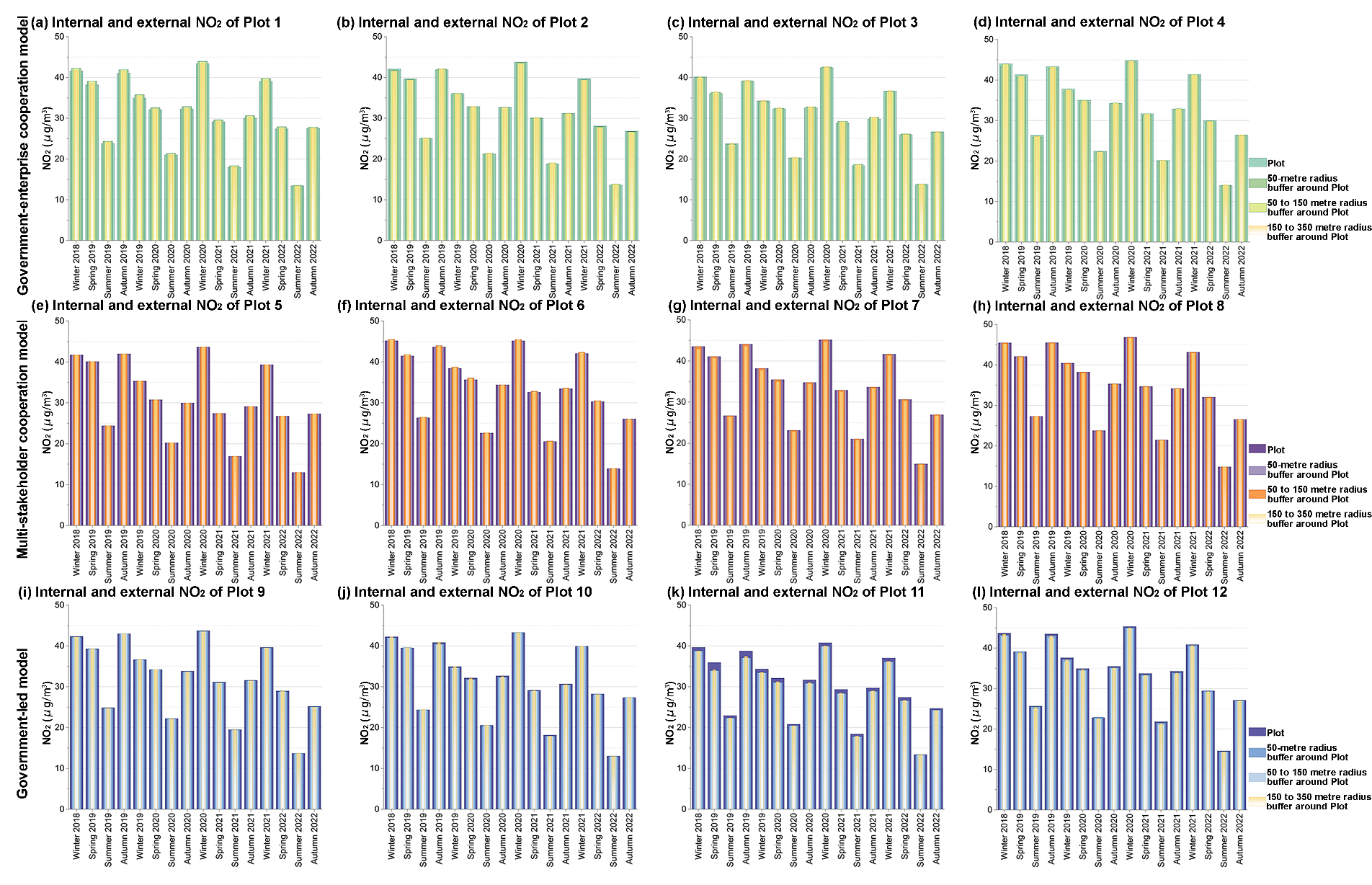


Supplementary Figure 10. NO_2_ concentration values within the plot and in three external buffer zones over a four-year period for the same plot.


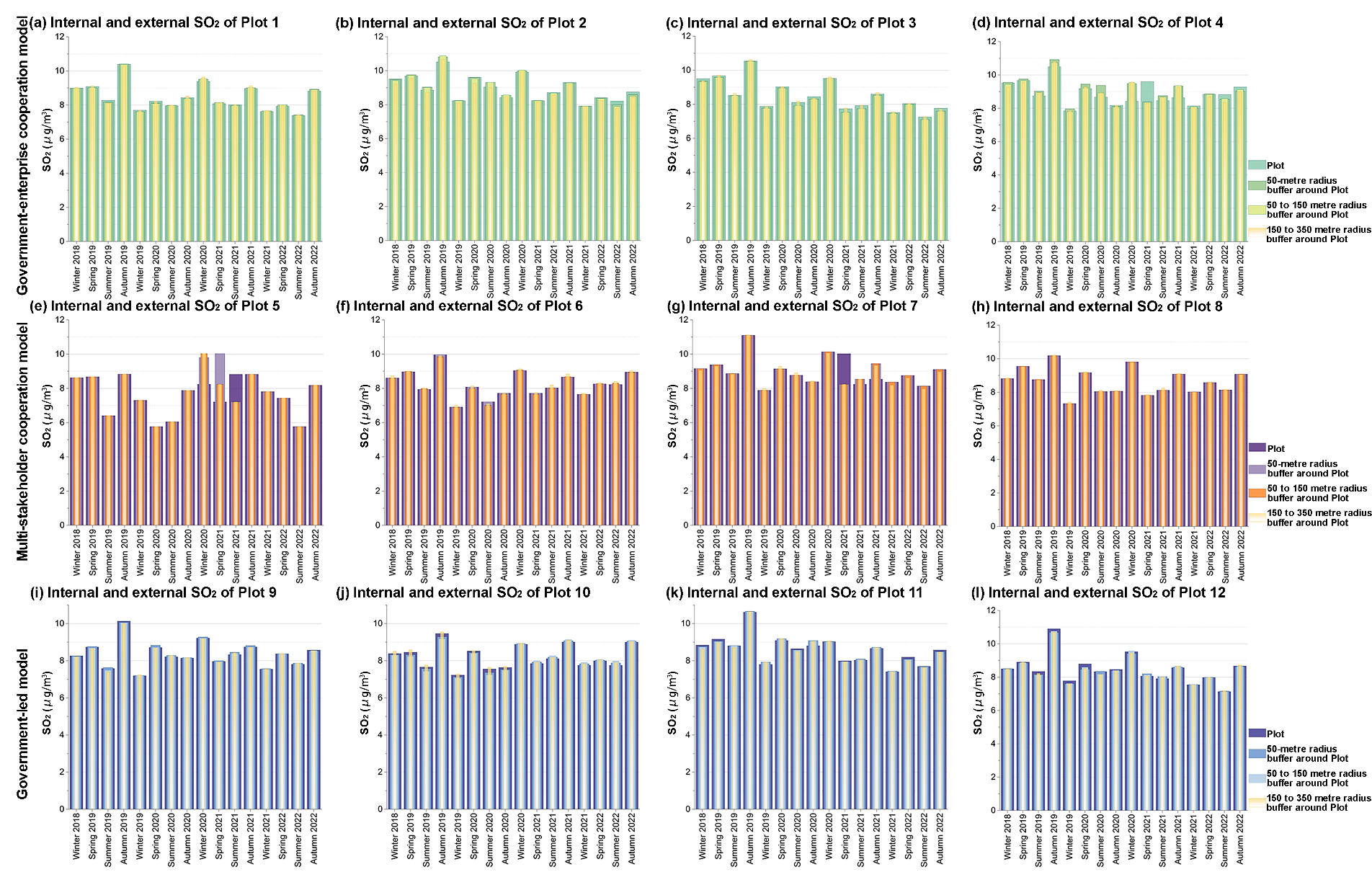


Supplementary Figure 11. SO_2_ concentration values within the plot and in three external buffer zones over a four-year period for the same plot.

Supplementary Table 1. The stages of urban regeneration corresponding to different plots over four years.

| Plot | The years corresponding to different stages of urban regeneration projects | |
| --- | --- | --- |
|  | Pre-regeneration stage | Regeneration implementation stage |
| Plot 1 | From 2018 to 2019 | From 2020 to 2022 |
| Plot 2 | From 2018 to 2020 | From 2021 to 2022 |
| Plot 3 | From 2018 to 2019 | From 2020 to 2022 |
| Plot 4 | From 2018 to 2020 | From 2021 to 2022 |
| Plot 5 | From 2018 to 2020 | From 2021 to 2022 |
| Plot 6 | From 2018 to 2020 | From 2021 to 2022 |
| Plot 7 | From 2018 to 2020 | From 2021 to 2022 |
| Plot 8 | From 2018 to 2019 | From 2020 to 2022 |
| Plot 9 | From 2018 to 2020 | From 2021 to 2022 |
| Plot 10 | From 2018 to 2020 | From 2021 to 2022 |
| Plot 11 | From 2018 to 2020 | From 2021 to 2022 |
| Plot 12 | From 2018 to 2020 | From 2021 to 2022 |

Supplementary Table 2. Changes in the differences of LST and Ta between inside and outside the plots after urban regeneration began (“－” indicates a decrease in temperature difference compared to before the regeneration started).

| Plot | Winter 2021 | | Spring 2022 | | Summer 2022 | | Autumn 2022 | |
| --- | --- | --- | --- | --- | --- | --- | --- | --- |
|  | LST difference | Air temperature difference | LST difference | Air temperature difference | LST difference | Air temperature difference | LST difference | Air temperature difference |
| Plot 1 | － | － |  |  |  |  |  |  |
| Plot 2 | － | － |  |  |  |  |  |  |
| Plot 3 | － | － |  | － |  | － |  | － |
| Plot 4 | － | － |  |  |  |  |  |  |
| Plot 5 |  |  |  |  | － | － | － |  |
| Plot 6 |  |  | － |  |  |  | － |  |
| Plot 7 |  | － |  |  |  |  | － |  |
| Plot 8 |  | － |  |  | － |  |  |  |
| Plot 9 |  |  |  | － |  |  | － | － |
| Plot 10 |  | － |  |  | － |  | － |  |
| Plot 11 | － | － |  | － |  |  | － |  |
| Plot 12 |  | － |  |  | － |  |  |  |

Supplementary Table 3. Changes in the differences of CO, NO_2_, and SO_2_ concentrations between inside and outside the plots after urban regeneration began (“－” indicates a decrease in the concentration difference compared to before the regeneration started, “＋” indicates an increase in the concentration difference compared to before the regeneration started).

| Plot | Winter 2021 | | | Spring 2022 | | | Summer 2022 | | | Autumn 2022 | | |
| --- | --- | --- | --- | --- | --- | --- | --- | --- | --- | --- | --- | --- |
|  | CO differ-ence | NO_2_ differ-ence | SO_2_ differ-ence | CO differ-ence | NO_2_ differ-ence | SO_2_ differ-ence | CO differ-ence | NO_2_ differ-ence | SO_2_ differ-ence | CO differ-ence | NO_2_ differ-ence | SO_2_ differ-ence |
| Plot 1 |  |  |  |  | － | － | － | － | － |  | － |  |
| Plot 2 |  | － | － |  |  |  | － |  |  | ＋ |  | － |
| Plot 3 | － |  | － | － | － | － |  | － |  |  |  |  |
| Plot 4 |  | － | － |  | － |  | － |  |  |  | － | － |
| Plot 5 |  |  |  |  |  |  |  |  |  |  |  |  |
| Plot 6 | － | － | － | － | － |  |  |  |  |  | － | － |
| Plot 7 |  | － |  |  | － |  |  |  |  |  | － |  |
| Plot 8 |  |  | － |  |  |  |  |  |  |  | － |  |
| Plot 9 | － | － | － | － | － | － |  | － | － |  |  | － |
| Plot 10 |  | － | － |  | － | － | － |  | － |  | － | － |
| Plot 11 |  |  | － |  | － |  |  | － |  | ＋ | － |  |
| Plot 12 | － | － |  | － | － | － | － | － | － |  | － | － |

Supplementary Table 4. Spatial gradient changes in LST and Ta outside the plots during the last four seasons after the start of urban regeneration (“↑” indicates a trend of higher temperatures with increasing distance from the center of the regeneration plot, “↓” indicates a trend of lower temperatures with increasing distance from the center of the regeneration plot).

| Plot | Winter 2021 | | Spring 2022 | | Summer 2022 | | Autumn 2022 | |
| --- | --- | --- | --- | --- | --- | --- | --- | --- |
|  | LST difference | Air temperature difference | LST difference | Air temperature difference | LST difference | Air temperature difference | LST difference | Air temperature difference |
| Plot 1 | ↓ | ↓ | ↓ |  | ↓ | ↓ | ↓ |  |
| Plot 2 | ↓ |  | ↓ | ↑ | ↓ |  | ↓ | ↑ |
| Plot 3 | ↓ |  | ↓ | ↓ | ↓ | ↓ |  | ↓ |
| Plot 4 |  | ↓ |  | ↓ |  | ↓ |  | ↓ |
| Plot 5 | ↑ | ↑ | ↑ | ↑ | ↑ | ↑ | ↑ |  |
| Plot 6 | ↑ | ↓ | ↑ | ↓ |  | ↓ | ↑ | ↓ |
| Plot 7 | ↓ |  | ↓ | ↓ | ↓ | ↑ | ↓ | ↑ |
| Plot 8 | ↓ | ↑ |  | ↑ |  | ↑ | ↓ |  |
| Plot 9 | ↓ |  | ↓ |  | ↓ |  | ↓ |  |
| Plot 10 | ↓ | ↓ | ↓ | ↓ | ↓ | ↓ | ↓ | ↓ |
| Plot 11 | ↓ |  | ↓ | ↑ | ↓ | ↑ | ↓ | ↑ |
| Plot 12 | ↑ |  | ↑ |  | ↑ |  | ↑ |  |

Supplementary Table 5. Spatial gradient changes in CO, NO_2_, and SO_2_ concentrations outside the plots during the last four seasons after the start of urban regeneration (“↑” indicates a trend of higher concentrations with increasing distance from the center of the regeneration plot, “↓” indicates a trend of lower concentrations with increasing distance from the center of the regeneration plot).

| Plot | Winter 2021 | | | Spring 2022 | | | Summer 2022 | | | Autumn 2022 | | |
| --- | --- | --- | --- | --- | --- | --- | --- | --- | --- | --- | --- | --- |
|  | CO differ-ence | NO_2_ differ-ence | SO_2_ differ-ence | CO differ-ence | NO_2_ differ-ence | SO_2_ differ-ence | CO differ-ence | NO_2_ differ-ence | SO_2_ differ-ence | CO differ-ence | NO_2_ differ-ence | SO_2_ differ-ence |
| Plot 1 | ↑ | ↓ |  | ↑ | ↓ | ↓ |  | ↓ |  | ↑ | ↓ | ↓ |
| Plot 2 | ↓ | ↓ | ↓ | ↓ | ↓ | ↓ |  | ↓ | ↓ |  | ↓ | ↓ |
| Plot 3 | ↓ | ↑ | ↑ |  |  | ↑ |  |  | ↑ | ↓ | ↑ | ↑ |
| Plot 4 |  |  | ↓ |  |  | ↓ |  |  | ↓ |  |  | ↓ |
| Plot 5 |  |  |  |  |  |  |  |  |  |  |  |  |
| Plot 6 |  | ↓ | ↑ |  | ↓ | ↑ |  |  | ↑ |  |  | ↑ |
| Plot 7 | ↓ | ↓ | ↓ | ↓ | ↓ | ↓ |  | ↓ | ↓ | ↓ | ↓ | ↓ |
| Plot 8 |  | ↓ | ↓ |  | ↓ |  |  | ↓ |  |  | ↓ | ↓ |
| Plot 9 | ↓ |  | ↓ | ↓ |  |  |  |  |  | ↓ |  |  |
| Plot 10 | ↓ | ↓ | ↓ | ↓ | ↓ | ↓ |  |  | ↓ | ↓ | ↓ | ↓ |
| Plot 11 |  | ↑ | ↓ |  | ↑ | ↓ |  | ↑ | ↓ |  |  | ↓ |
| Plot 12 | ↓ | ↑ | ↓ | ↓ | ↑ |  |  | ↑ |  | ↓ | ↑ |  |

Supplementary Table 6. Impact of urban regeneration models led by different stakeholders on LST.

|  |  | Inside the plot | | | | | | Outside the plot | |
| --- | --- | --- | --- | --- | --- | --- | --- | --- | --- |
| Urban regeneration model | Plot | Maximum LST fluctuatio-ns | Minimal LST fluctuation-s | Lower LST in winter 2021 | Lower LST in spring 2022 | Lower LST in summer 2022 | Lower LST in autumn 2021 | LST inside the plot are higher than in the outer buffer zone | Lower LST at greater diatances from the buffer zone |
| Government-enterprise cooperation model | Plot 1 | √ |  |  | √ |  |  |  | √ |
|  | Plot 2 | √ |  |  | √ |  |  |  | √ |
|  | Plot 3 | √ |  |  | √ |  |  |  | √ |
|  | Plot 4 | √ |  |  | √ |  | √ | √ | √ |
| Multi-stakeholder cooperation model | Plot 5 |  |  | √ | √ | √ |  |  |  |
|  | Plot 6 |  |  |  |  |  |  |  |  |
|  | Plot 7 |  |  |  |  |  |  |  |  |
|  | Plot 8 |  |  |  |  |  |  | √ |  |
| Government-led model | Plot 9 |  | √ | √ |  | √ | √ | √ | √ |
|  | Plot 10 |  | √ | √ |  | √ | √ | √ | √ |
|  | Plot 11 |  | √ | √ | √ | √ | √ | √ | √ |
|  | Plot 12 |  | √ | √ |  | √ | √ |  |  |

Supplementary Table 7. Impact of urban regeneration models led by different stakeholders on Ta.

|  |  | Inside the plot | | | | | | Outside the plot | |
| --- | --- | --- | --- | --- | --- | --- | --- | --- | --- |
| Urban regeneration model | Plot | Maximum Ta fluctuations | Minimal Ta fluctuations | Lower Ta in winter 2021 | Lower Ta in spring 2022 | Lower Ta in summer 2022 | Lower Ta in autumn 2021 | Ta inside the plot are higher than in the outer buffer zone | Lower Ta at greater diatances from the buffer zone |
| Government-enterprise cooperation model | Plot 1 |  |  |  |  |  |  | √ |  |
|  | Plot 2 |  |  |  |  |  |  |  |  |
|  | Plot 3 |  |  |  |  |  |  |  | √ |
|  | Plot 4 |  |  |  |  |  |  |  | √ |
| Multi-stakeholder cooperation model | Plot 5 |  |  |  |  | √ | √ |  |  |
|  | Plot 6 |  |  |  |  | √ | √ | √ | √ |
|  | Plot 7 |  |  |  |  | √ | √ | √ |  |
|  | Plot 8 |  |  |  |  | √ | √ |  |  |
| Government-led model | Plot 9 |  |  | √ | √ |  |  |  |  |
|  | Plot 10 |  |  | √ | √ |  |  |  | √ |
|  | Plot 11 |  |  | √ | √ |  |  |  |  |
|  | Plot 12 |  |  | √ | √ |  |  | √ |  |

Supplementary Table 8. Impact of urban regeneration models led by different stakeholders on CO concentration.

|  |  | Inside the plot | | | | | | Outside the plot | |
| --- | --- | --- | --- | --- | --- | --- | --- | --- | --- |
| Urban regeneration model | Plot | Maximum CO fluctuations | Minimal CO fluctuations | Lower CO in winter 2021 | Lower CO in spring 2022 | Lower CO in summer 2022 | Lower CO in autumn 2021 | CO inside the plot are higher than in the outer buffer zone | Lower CO at greater diatances from the buffer zone |
| Government-enterprise cooperation model | Plot 1 |  | √ | √ |  |  |  |  |  |
|  | Plot 2 |  | √ | √ |  |  |  | √ | √ |
|  | Plot 3 |  | √ | √ |  |  |  |  | √ |
|  | Plot 4 |  | √ | √ |  |  |  | √ |  |
| Multi-stakeholder cooperation model | Plot 5 |  |  |  | √ | √ |  |  |  |
|  | Plot 6 |  |  |  | √ | √ |  |  |  |
|  | Plot 7 |  |  |  | √ | √ |  |  | √ |
|  | Plot 8 |  |  |  | √ | √ |  |  |  |
| Government-led model | Plot 9 | √ |  |  |  |  | √ | √ | √ |
|  | Plot 10 | √ |  |  |  |  | √ | √ | √ |
|  | Plot 11 | √ |  |  |  |  | √ | √ | √ |
|  | Plot 12 | √ |  |  |  |  | √ | √ | √ |

Supplementary Table 9. Impact of urban regeneration models led by different stakeholders on NO_2_ concentration.

|  |  | Inside the plot | | | | | | Outside the plot | |
| --- | --- | --- | --- | --- | --- | --- | --- | --- | --- |
| Urban regeneration model | Plot | Maximum NO_2_ fluctuations | Minimal NO_2_ fluctuations | Lower NO_2_ in winter 2021 | Lower NO_2_ in spring 2022 | Lower NO_2_ in summer 2022 | Lower NO_2_ in autumn 2021 | NO_2_ inside the plot are higher than in the outer buffer zone | Lower NO_2_ at greater diatances from the buffer zone |
| Government-enterprise cooperation model | Plot 1 |  | √ |  | √ | √ |  |  | √ |
|  | Plot 2 |  | √ |  | √ | √ |  |  | √ |
|  | Plot 3 |  | √ |  | √ | √ |  |  |  |
|  | Plot 4 |  | √ |  | √ | √ |  |  |  |
| Multi-stakeholder cooperation model | Plot 5 | √ |  | √ | √ | √ |  | √ |  |
|  | Plot 6 | √ |  |  |  |  |  | √ |  |
|  | Plot 7 | √ |  |  |  |  |  | √ | √ |
|  | Plot 8 | √ |  |  |  |  |  | √ | √ |
| Government-led model | Plot 9 |  |  | √ |  |  | √ | √ |  |
|  | Plot 10 |  |  | √ |  |  | √ |  |  |
|  | Plot 11 |  |  | √ | √ |  | √ | √ |  |
|  | Plot 12 |  |  | √ |  |  | √ | √ |  |

Supplementary Table 10. Impact of urban regeneration models led by different stakeholders on SO_2_ concentration.

|  |  | Inside the plot | | | | | | Outside the plot | |
| --- | --- | --- | --- | --- | --- | --- | --- | --- | --- |
| Urban regeneration model | Plot | Maximum SO_2_ fluctuations | Minimal SO_2_ fluctuations | Lower SO_2_ in winter 2021 | Lower SO_2_ in spring 2022 | Lower SO_2_ in summer 2022 | Lower SO_2_ in autumn 2021 | SO_2_ inside the plot are higher than in the outer buffer zone | Lower SO_2_ at greater diatances from the buffer zone |
| Government-enterprise cooperation model | Plot 1 |  | √ |  |  |  |  |  | √ |
|  | Plot 2 |  | √ |  |  |  |  |  | √ |
|  | Plot 3 |  | √ |  |  |  |  |  |  |
|  | Plot 4 |  | √ |  |  |  |  |  | √ |
| Multi-stakeholder cooperation model | Plot 5 | √ |  |  |  |  |  |  |  |
|  | Plot 6 | √ |  |  |  |  |  |  |  |
|  | Plot 7 | √ |  |  |  | √ |  |  | √ |
|  | Plot 8 | √ |  |  |  |  |  |  |  |
| Government-led model | Plot 9 |  | √ | √ | √ | √ | √ |  | √ |
|  | Plot 10 |  | √ | √ | √ | √ | √ |  | √ |
|  | Plot 11 |  | √ | √ | √ | √ | √ |  | √ |
|  | Plot 12 |  | √ | √ | √ | √ | √ |  | √ |
